# Pch2 Links Chromosome Axis Remodeling at Future Crossover Sites and Crossover Distribution during Yeast Meiosis

**DOI:** 10.1371/journal.pgen.1000557

**Published:** 2009-07-24

**Authors:** Neeraj Joshi, Aekam Barot, Christine Jamison, G. Valentin Börner

**Affiliations:** Center for Gene Regulation in Health and Disease, Department of Biological, Geological, and Environmental Sciences, Cleveland State University, Cleveland, Ohio, United States of America; The University of North Carolina at Chapel Hill, United States of America

## Abstract

Segregation of homologous chromosomes during meiosis I depends on appropriately positioned crossovers/chiasmata. Crossover assurance ensures at least one crossover per homolog pair, while interference reduces double crossovers. Here, we have investigated the interplay between chromosome axis morphogenesis and non-random crossover placement. We demonstrate that chromosome axes are structurally modified at future crossover sites as indicated by correspondence between crossover designation marker Zip3 and domains enriched for axis ensemble Hop1/Red1. This association is first detected at the zygotene stage, persists until double Holliday junction resolution, and is controlled by the conserved AAA+ ATPase Pch2. Pch2 further mediates crossover interference, although it is dispensable for crossover formation at normal levels. Thus, interference appears to be superimposed on underlying mechanisms of crossover formation. When recombination-initiating DSBs are reduced, Pch2 is also required for viable spore formation, consistent with further functions in chiasma formation. *pch2Δ* mutant defects in crossover interference and spore viability at reduced DSB levels are oppositely modulated by temperature, suggesting contributions of two separable pathways to crossover control. Roles of Pch2 in controlling both chromosome axis morphogenesis and crossover placement suggest linkage between these processes. Pch2 is proposed to reorganize chromosome axes into a tiling array of long-range crossover control modules, resulting in chiasma formation at minimum levels and with maximum spacing.

## Author Summary

In the germ line of sexually reproducing organisms, haploid gametes are generated from diploid precursor cells by a specialized cell division called meiosis. Reduction by half of chromosome numbers during the first meiotic division depends on genetic exchange, resulting in the formation of crossovers. Without crossovers, pairs of homologous chromosomes frequently fail to separate, resulting in unbalanced gametes with a surplus or deficit of individual chromosomes. Along a given chromosome, crossovers form in different locations in different cells, but distribution of crossovers within each cell is controlled in two ways: first, at least one crossover is formed along each homolog pair, irrespective of size; second, a crossover in a given interval reduces the frequency of crossovers in adjacent chromosome regions. Here, we identify functions of the evolutionarily conserved protein Pch2 in suppressing additional crossovers in adjacent regions and ensuring homolog segregation under certain conditions. Pch2 further controls the assembly of chromosome axis protein Hop1 at future crossover sites. Our findings reveal that chromosome axes undergo structural changes at the same positions where crossovers occur. Thus, axis remodeling and crossover placement are linked via Pch2.

## Introduction

During meiosis, a single round of DNA replication is followed by two rounds of chromosome separation, with homologous chromosomes (homologs) segregating during meiosis I and sister chromatids during meiosis II. Homolog segregation critically depends on formation of crossovers (COs) between homologs. COs, cytologically detectable as chiasmata, in combination with sister chromatid connections, mediate the correct positioning of homolog pairs in the meiosis I spindle. Without COs, homologs frequently fail to segregate, resulting in formation of aneuploid gametes, i.e. gametes with a chromosome surplus or deficit. Aneuploid gametes are one of the major causes for stillbirths and birth defects in humans [1].

CO formation occurs via a carefully orchestrated program during prophase of meiosis I entails close homolog juxtaposition, followed by reciprocal exchange of chromosome arms through homologous recombination [2]. On the DNA level, meiotic recombination is initiated by formation of programmed double strand breaks (DSBs) at multiple genome positions [3]–[5]. A non-random subset of DSBs undergoes stable interaction with a homologous chromatid, giving rise to COs, while the remainder of DSBs progress to alternative fates, including non-crossovers (NCOs), i.e. recombination events without exchange of flanking chromosome arms, as well as repair events with the sister chromatid [6]–[8].

Studies in fungi including *S. cerevisiae* have provided an understanding of meiotic recombination at the molecular level. Processing of meiotically induced DSBs depends on numerous proteins with related roles in mitotic DSB repair, but there are also prominent differences between these processes: First, during meiosis, homologs rather than sister chromatids serve as partners for homologous recombination [6]. Second, CO formation is enhanced over that of NCOs [7]. Following 5′ resection, DSBs undergo strand invasion of intact non-sister homologous chromatids. Pathways leading to COs and NCOs appear to bifurcate no later than the stage of strand invasion: Single end invasions (SEIs) emerge as the first CO-specific intermediate, subsequently giving rise to double Holliday junctions which are specifically resolved as COs [9]–[11]. NCOs likely arise via an alternative pathway characterized by a more transient strand invasion [7]. Notably, only COs provide interhomolog connections as required for homolog segregation.

Recombination is temporally and spatially coordinated with dramatic changes in global chromosome structure culminating in the assembly of the synaptonemal complex (SC). The SC, a widely conserved proteinaceous structure, stably juxtaposes homologs along their entire lengths during the pachytene stage [12]. SC formation is initiated during the leptotene stage when axial elements first form between and along sister chromatids. During the zygotene stage, axial elements of homologous chromosomes become closely juxtaposed via the SC central element which starts polymerizing from discrete sites; achieving full length homolog synapsis during the pachytene stage. Recombination is initiated via induction of DSBs during the leptotene stage, followed by onset of strand invasion at the transition from the leptotene to the zygotene stage [9]. During the pachytene stage, in the context of fully formed SC, double Holliday junctions are formed and resolved into COs, with NCOs emerging somewhat earlier [9]–[11].

Morphogenesis of the SC and recombination are highly interdependent, as indicated by (i) requirements for recombination proteins for SC assembly, and (ii) functions of SC components in recombination. In *S. cerevisiae*, DSBs are introduced by the widely conserved topoisomerase homolog Spo11 [5]. Spo11-dependent DSB formation is also required for SC assembly. Prominent components of yeast axial elements include Hop1 and its binding partner Red1, as well as meiosis-specific cohesin Rec8 and cohesin-associated proteins, e.g. Spo76/Pds5 [13]–[16]. Hop1 and Red1 further mediate normal DSB formation and preferential interaction of DSBs with homologs rather than sister chromatids [6], [17]–[19]. Zip1 is a prominent component of the SC central element. Zip1 starts polymerizing from both centromeres and from positions of designated CO sites [20]–[24]. Prior to assembly into full length SC, Zip1 mediates timely and efficient CO-specific strand invasion during recombination [11]. Two ZMM proteins, Zip2 and Zip3, are required for formation of most COs and also mediate normal SC assembly. Zip3 is present along fully formed SC with the number and distribution expected for CO designated sites, in *S. cerevisiae* and *C.elegans* [21],[22],[25],[26]. Finally, regions surrounding emerging COs are structurally modified as suggested by localized separation of sister chromatids at sites of ongoing recombination [16]. Later, when chiasmata emerge, they are characterized by extended regions of sister axis separation flanking the position of an established CO (see example in ref. [27]).

CO placement along homolog pairs is non-random at several levels: First, CO assurance guarantees formation of at least one CO per bivalent (e.g. ref. [28]). Second, CO homeostasis enhances CO formation at the expense of NCOs when initiating DSBs are artificially reduced [29]. Third, CO interference reduces the frequency of COs in regions adjacent to established COs resulting in maximally spaced COs [30].

The three levels of CO control indicate communication along chromosomes between sites of ongoing recombination. CO interference reduces CO frequencies over large physical distances, >100 kb in yeast and >100 Mb in higher eukaryotes [8],[31]. CO assurance and CO homeostasis suggest mechanism(s) that sense overall CO and/or DSB levels, affecting the outcome of ongoing recombination events. Timing, mechanism and the functional relationship between CO control and meiotic recombination pathway(s) are poorly understood. CO control is thought to operate on randomly distributed recombination interactions, a non-random subset of which become designated as future COs with the remainder progressing to NCOs. CO designation likely occurs no later than zygotene, as suggested by occurrence of cytological markers of CO-designation at this stage, and by concurrent appearance of CO specific recombination intermediates [11]. Linkage between CO assurance and CO interference was inferred from coordinate loss or retention of both features in certain mutant situations [29],[32]. Conversely, CO interference is retained in two *zmm* mutants (*zip4Δ*, *spo16Δ*) despite apparent loss of CO assurance, indicating that separable pathways contribute to CO control [28]. Structural chromosome components responsible for CO control also remain unknown. Normal interference distribution of CO-designation marker Zip2 in *zip1Δ* suggests that the SC central element is not required for crossover interference [22]. Notably, in *zip1Δ*, CO designation sites/Zip2 foci exhibit interference distribution, while CO interference is defective, indicating uncoupling between chromosome morphogenesis and events on the DNA level [22].

The widely conserved AAA+ ATPase Pch2 performs important functions in cell cycle control, recombination and chromosome morphogenesis during mutant and WT meiosis. Identified as a yeast mutant that bypasses meiotic arrest in *zip1Δ*, Pch2 also mediates mutant delay/arrest in *C. elegans* and *Drosophila* [33]–[38]. During yeast WT meiosis, Pch2 mediates timely resolution of double Holliday junctions and formation of COs and NCOs [35],[37]. Processing of a subset of recombination intermediates also depends on Pch2 in mouse [36]. In yeast, Pch2 further mediates assembly of structurally normal SC, controlling installation of axis component Hop1 and SC central element protein Zip1 along meiotic chromosomes in a pattern of alternating hyperabundance [37]. This pattern likely arises due to uniform loading of Hop1 and Zip1 at base levels along the length of the SC, corresponding to the uniform appearance of the SC detected by electron microscopy, in combination with additional domainal loading of either protein. Absence of Pch2 results in uniform localization patterns of Hop1 and Zip1 along the length of meiotic chromosomes [37].

Here, we have investigated the interplay between meiotic chromosome morphogenesis and CO control in yeast. We demonstrate intimate coordination between controlled CO distribution and axial element morphogenesis, as suggested by frequent association between Zip3-marked CO-designation sites and domains of preferential Hop1/Red1 loading. Association between Zip3 and Hop1/Red1 becomes detectable prior to substantial SC polymerization, consistent with axis differentiation at future CO sites early during meiosis. Furthermore, Hop1-Zip3 association is detected in *ndt80Δ*-arrested cells indicating its establishment independent of and prior to double Holliday junction resolution. Pch2 controls chromosome axis status by (i) specifying amount and pattern of chromosomal Hop1, (ii) limiting Zip3 positions along pachytene chromosomes and (iii) mediating global axis shortening. While competent for CO formation at normal levels, *pch2Δ* is defective in controlling the distribution of COs along chromosome arms. In *pch2Δ*, (i) CO interference is defective, and (ii) spore viability is drastically reduced upon global reduction of initiating DSBs. The *pch2Δ* phenotype is dramatically modulated by incubation conditions, including temperature, suggesting the existence of Pch2-independent back-up systems for crossover interference and for maintenance of normal spore viability despite reduced DSB levels. We propose a model where Pch2 mediates establishment of multiple CO control modules along each chromosome, with potential effects on CO interference and chiasma function.

## Results

### Pch2 localizes to chromosome arms during the early zygotene stage

Pch2 mediates domainal hyperabundance of axis protein Hop1 along pachytene chromosomes [37]. Loss of domain structure in *pch2Δ* during zygotene suggests Pch2 functions at or before this stage. To examine Pch2 localization throughout meiosis I prophase, an isogenic SK1 strain homozygous for N-terminally 3×HA-tagged Pch2 ( = HA-Pch2) was induced to undergo synchronized meiosis at 33°C. The 3×HA-tagged Pch2 construct used here complements Pch2 function as suggested by its ability to confer arrest in *zip1Δ*. It is identical to a construct previously examined in a different strain background (data not shown, A. Hochwagen, personal communication, see Materials and Methods for details; [33]).

**Figure 1:**
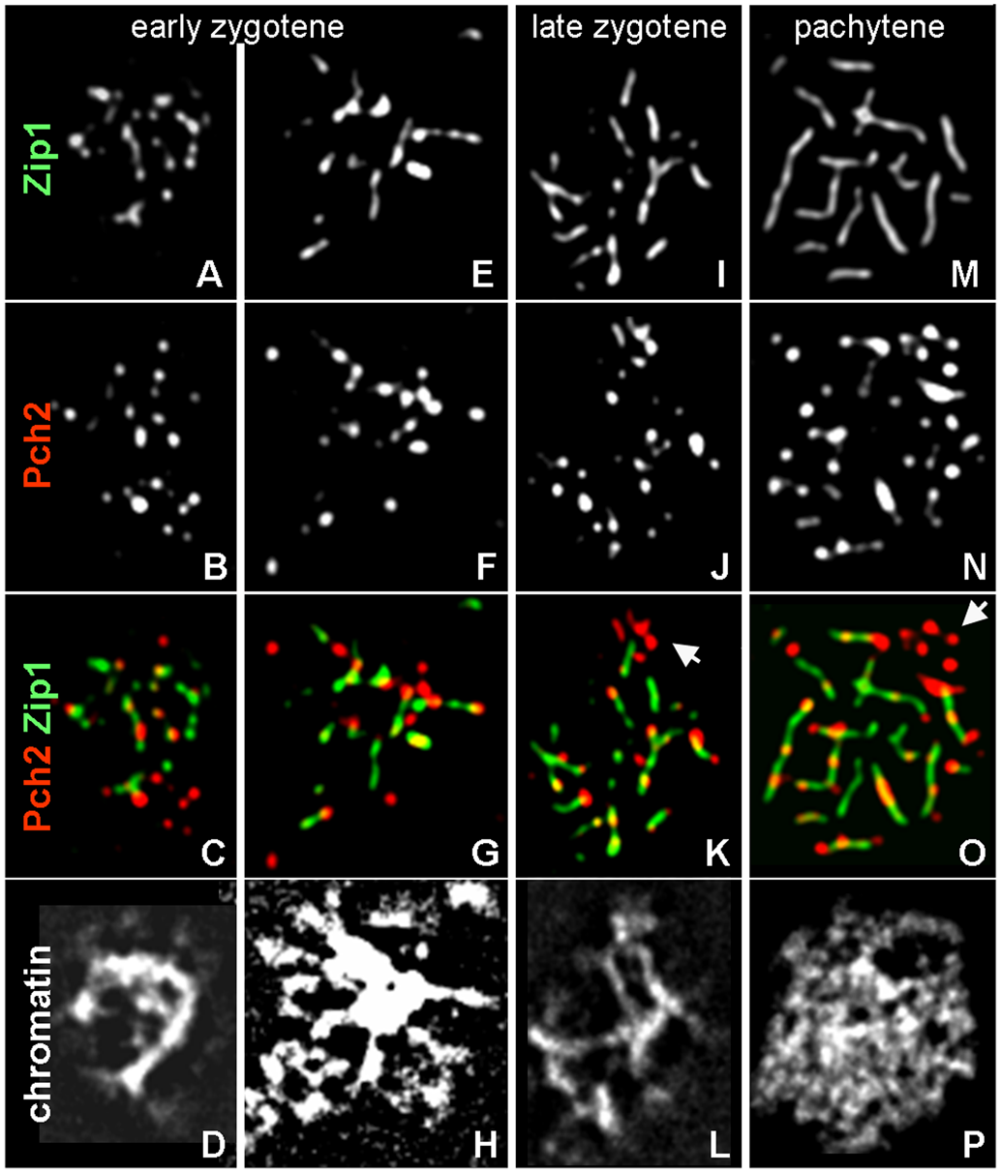
Association of Pch2 with WT chromosomes at different stages of SC polymerization. (A–H) Early zygotene nuclei; Zip1 (A,E), and HA-Pch2 (B,F). (I–L) Late zygotene nucleus. (M–P) Pachytene nucleus. Arrows indicate rDNA clusters, identified by Pch2 localization pattern and lack of Zip1 (K,O). Spread zygotene and pachytene nuclei are identified base on extend of Zip1 staining. Colors are indicated by the corresponding labels. Meiosis was induced at 33°C.

Pch2 localization was examined at all stages of meiosis I prophase. At specified time points, cells were surface spread and immunodecorated with antibodies against the HA-epitope, and SC central element component Zip1 [20]. Cells progressed through meiosis with appropriate timing [11]: Late leptotene nuclei, containing <10 Zip1 staining foci, first appear at 2 hrs (Figure S1). Zygotene nuclei carrying multiple Zip1 foci (“early zygotene”) and/or Zip1 in partial lines (“late zygotene”) are prominent at 4 to 5 hrs (Figure 1A, 1AE, and 1I). Pachytene cells exhibiting mostly continuous lines of Zip1 along most of the 16 homolog pairs reach peak levels between 4 to 7 hours and disappear shortly before the onset of nuclear divisions (Figure 1M and Figure S1).

In pachytene nuclei, multiple Pch2 foci of comparable intensities are detected on most of the chromatin mass (Figure 1M–1P). The majority of Pch2 foci is associated with Zip1, but cells frequently also contain ∼five grouped foci in a crescent-shaped, Zip1-free chromatin region, likely corresponding to the nucleolus (Figure 1K and 1O; see ref. [33]). In pachytene nuclei, 21 (±7 S.D.) Pch2 foci residing outside the nucleolus (referred to as chromosomal Pch2 hereafter) are detected (n = 149 nuclei; Figure 1O). Pch2 is also present at chromosomal and presumed nucleolar positions in early and late zygotene nuclei where 16 (±5 S.D.) Pch2 foci are detected (n = 25 nuclei), some of which localize to several Zip1-free regions, suggesting localization to unsynapsed chromosomes. Together, these data indicate that Pch2 starts localizing abundantly to chromosome arms during the early zygotene stage, reaching maximum levels during the pachytene stage. Nucleolar and chromosomal Pch2 staining exhibits comparable intensities here, yet appears more prominent in the nucleolus in an earlier report [33]. Such differences could be due to effects of different spreading protocols and/or imaging systems.

### Pch2 localizes to crossover-designated sites

Pch2 promotes timely formation of recombination products [37], and plays roles in CO control (see below). Zip3 is a cytological marker for CO-designated sites, forming interference-distributed foci along pachytene chromosomes with numbers corresponding to COs [22]. To examine localization of chromosomal Pch2 with respect to ongoing recombination interactions, meiosis was induced in strains homozygous for HA-Pch2 and C-terminally GFP-tagged Zip3. (*ZIP3-GFP* complements *ZIP3* function as suggested by spore viabilities >85%, normal CO levels by physical analysis and WT-like progression through meiosis (G.V.B. and O. Nanassy, unpublished)).

**Figure 2:**
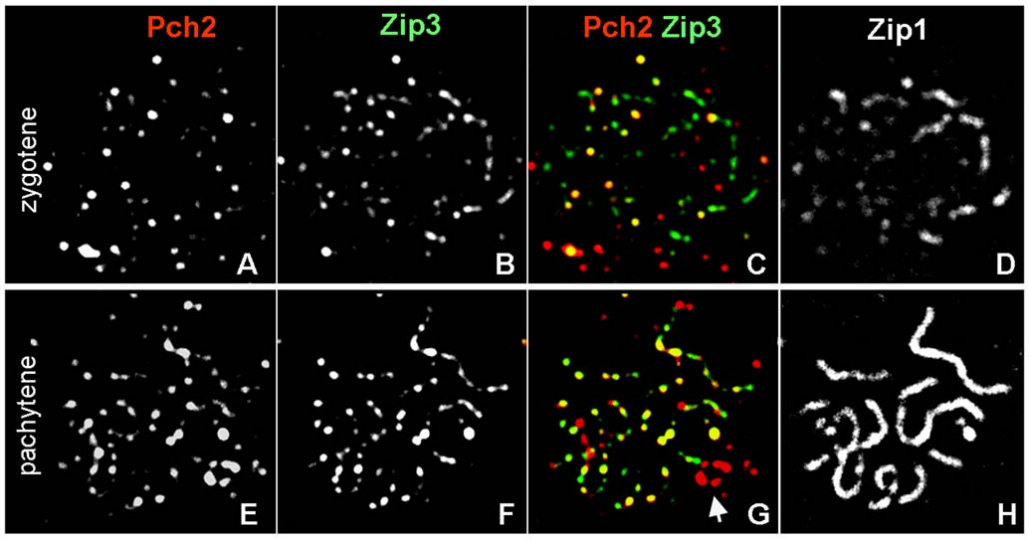
Association of Pch2 with CO designation marker Zip3 at different stages of synapsis. (A–D) Spread nucleus during early zygonema from a strain carrying HA-Pch2 and Zip3-GFP, stained with antibodies against HA- and GFP-epitopes as well as Zip1. (E–H) Spread pachytene nucleus. Arrow indicates rDNA cluster (G). Colors are indicated by the corresponding labels. Meiosis was induced at 33°C.

Cells from a synchronous time course carried out at 33°C were spread and stained with appropriate antibodies. Anti-Zip1 antibody was used to determine stages of cells. Number and localization patterns of Zip3 in zygotene and pachytene nuclei correspond well with earlier reports [21]–[23]. Pachytene nuclei contain 61 (±6 S.D.) Zip3 and 31 (±12 S.D.) Pch2 foci (n = 42 nuclei; see Figure 2E–2H). Importantly, a substantial number of Pch2 foci colocalizes with Zip3: In pachytene nuclei, 54% (±17% S.D.) of Pch2 foci are associated with Zip3 foci, compared to 18% (±10% S.D.) fortuitous colocalization (n = 17 nuclei; see Materials and Methods for details on analysis of fortuitous colocalization). Colocalization of Pch2 with Zip3 is also observed in zygotene nuclei where 44 (±16 S.D.) Zip3 and 25 (±12 S.D.) Pch2 foci are detected (n = 28 nuclei; Figure 2A–2D). Of the Pch2 foci detected, 58% (±18% S.D.) colocalize with Zip3, compared to 13% (±10% S.D.) fortuitous colocalization (n = 13 nuclei). Similar localization patterns are observed in the same strain incubated at 30°C (data not shown). Together, these data demonstrate that chromosomal Pch2 partially and/or transiently associates with Zip3-marked CO-designated sites. This association could be related to Pch2's function in CO placement and/or CO-associated domain organization (see below).

### Association between Hop1/Red1 hyperabundance domains and crossover-designated sites

**Figure 3:**
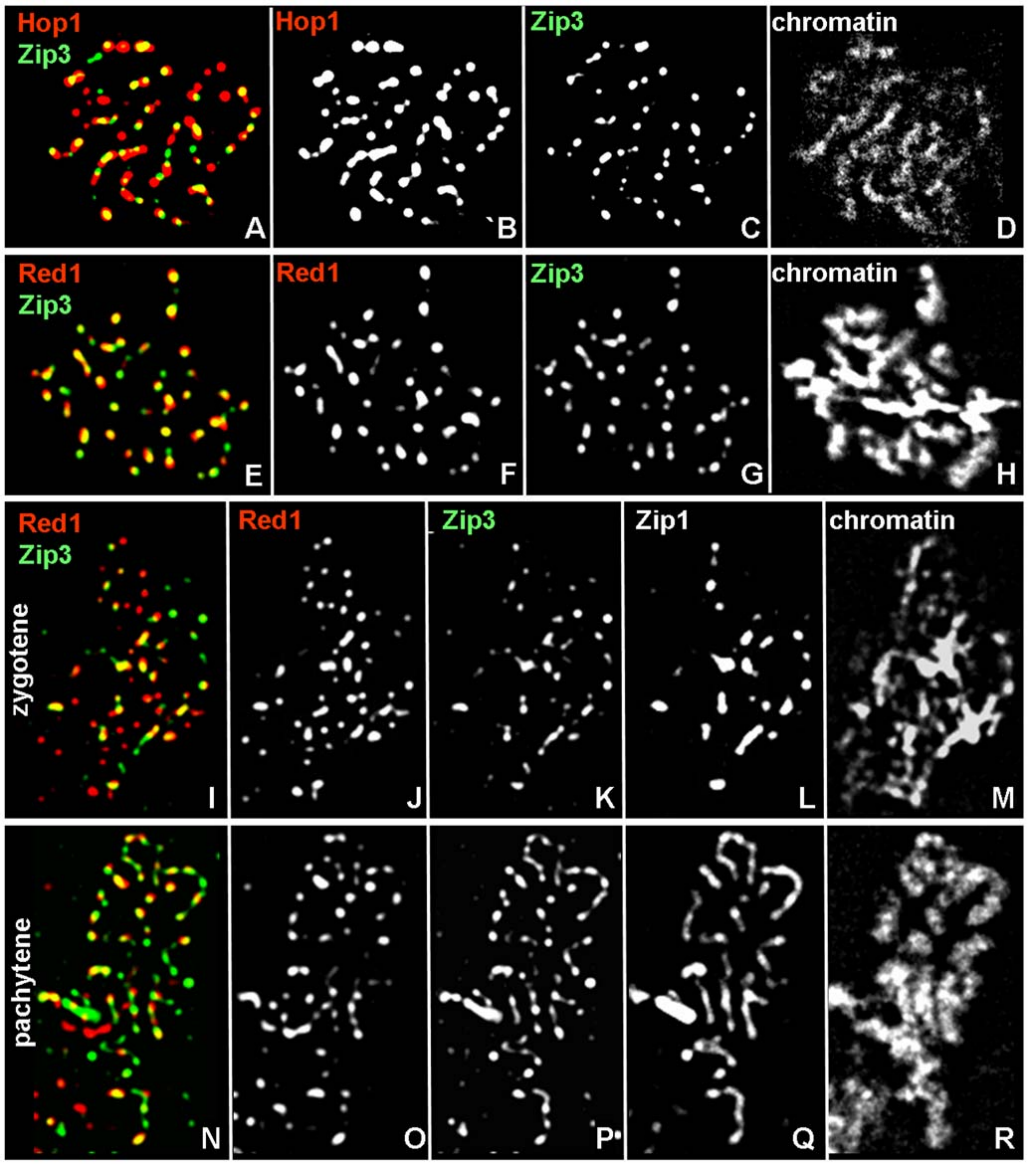
Association of axis components Hop1 and Red1 with CO–designation marker Zip3 at different stages of synapsis. (A–D) Spread pachytene nucleus stained with antibodies against Hop1 and Zip3-GFP. (E–H) Spread pachytene nucleus labeled with antibodies against Red1-HA and Zip3-GFP. (I–R) Spread nuclei from time course shown in (E–H) stained with antibody against Zip1 as well as Red1-HA and Zip3-GFP. (I–M) Spread zygotene nucleus. (N–R) Spread pachytene nucleus. Colors are indicated by the corresponding labels. Yellow colors in (A,E,I,N) indicate overlap between the indicated protein signals. Meiosis was induced at 33°C.

To gain insights into positional identities of Hop1-enriched axis domains, Zip3 and Hop1 localization were examined in a synchronous WT time course at a time when pachytene cells are abundant [11]. In WT, at t = 7 hrs, >50% of undivided nuclei are at the pachytene stage, as indicated by Zip1 staining patterns (data not shown). In the same cell population, Hop1 and Zip3 localization are remarkably similar in number and position: Hop1 localizes to 55 (±13 S.D.) foci while Zip3-GFP localizes to 56 (±13 S.D.) foci per nucleus (Figure 3A–3D; n = 68 nuclei). When Hop1 and Zip3 localization patterns in the same nuclei are compared, a striking correspondence in position emerges: 72% (±10% S.D.) of Zip3 foci colocalize with Hop1, and 73% (±15% S.D.) of Hop1 foci colocalize with Zip3. Fortuitous colocalization in the same nuclei is 17% (±7% S.D.) and 17% (±8% S.D.), respectively. These results suggest that CO-designated recombination interactions frequently localize to chromosome domains enriched for Hop1.

To examine Zip3 localization relative to another axis protein and to determine the stage of meiosis in the same cells, spread nuclei were triple-stained for Red1, Zip3, and Zip1 in a strain homozygous for C-terminally HA-tagged Red1 (Red1-HA) [17] and Zip3-GFP. High levels of colocalization between Zip3 and Red1 were observed at both the zygotene and pachytene stages (Figure 3E–3H). In zygotene nuclei, 57 (±16 S.D.) Red1 foci and 50 (±19 S.D.) Zip3 foci are detected: 65% (±10% S.D.) of Zip3 foci colocalize with Red1, and 55% (±14% S.D.) of Red1 foci colocalize with Zip3 (n = 72 nuclei; Figure 3I–3M). In pachytene nuclei, Red1 localizes to 53 (±11 S.D.) foci, and Zip3 to 55 (±15 S.D.) foci; 59% (±10% S.D.) Zip3 foci colocalize with Red1, and 60% (±14% S.D.) Red1 foci colocalize with Zip3 (n = 57 nuclei; Figure 3N–3R). Thus, Red1 is also frequently associated with chromosome regions designated to undergo CO formation.

Together, these results have two key implications: Association of Zip3 and Hop1/Red1 (i) at the same sites along pachytene chromosomes suggests spatial linkage between Hop1-enriched domains and CO placement, and (ii) temporal coincidence with CO/NCO differentiation during the zygotene stage [10],[11].

We note that not all Zip3 foci are associated with Hop1/Red1 in every cell. This association may be transient and/or only a subset of Zip3 associates with Red1/Hop1. Furthermore, Zip3 occupies presumed CO designation sites only during the pachytene stage, while it localizes to centromeres in pre-zygotene cells [22],[24]. In pre-zygotene cells, Zip3 is detected at small numbers and rarely colocalizes with abundantly staining Hop1 or Red1 (data not shown).

### Normal association between Zip3 and Hop1 depends on Dmc1, but not on Ndt80

We next investigated Hop1 and Zip3 localization in *dmc1Δ* and *ndt80Δ*, two meiotic mutants exhibiting distinct recombination blocks: In the absence of Rad51-paralog Dmc1, hyperresected DSBs accumulate, and COs and NCOs are eliminated, consistent with a role of Dmc1 in strand invasion (G.V.B., unpublished data; ref. [39]). Without transcription factor Ndt80, NCOs appear normally, but double Holliday junctions accumulate and COs are reduced accordingly [10]. Cells further arrest in *ndt80Δ* at a *bona fide* normal pachytene stage, as suggested by formation of viable spores upon induction of Ndt80 [40].

**Figure 4:**
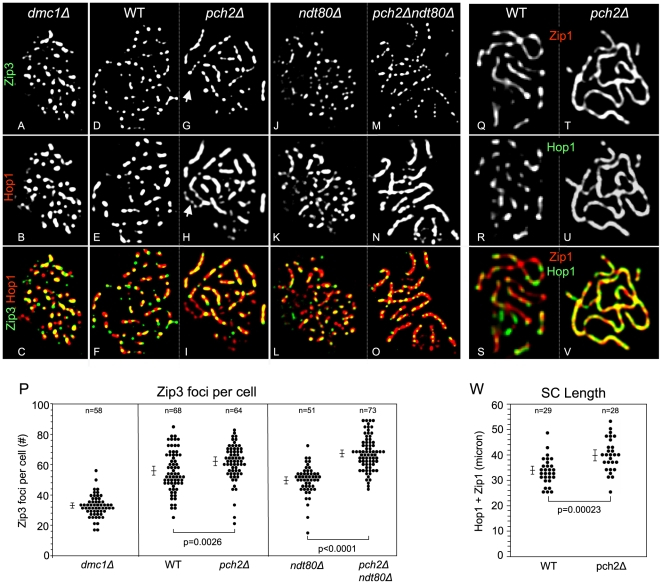
Effects of Dmc1, Pch2, and Ndt80 on Zip3 and Hop1 localization and SC length in WT and *pch2Δ*. (A–P) Spread nuclei from *dmc1Δ* (A–C), WT (*PCH2*) (D–F), *pch2Δ* (G–I), *PCH2ndt80Δ* (J–L), *pch2Δndt80Δ* (M–O) were stained with antibodies against Hop1 and Zip3. Colors are indicated by the corresponding labels in the individual channels. Regions of overlap between Zip3 and Hop1 are indicated by yellow color. Note the patchy versus continuous Hop1 localization in WT (E,K) versus *pch2Δ* (H,N). White arrow in (H) indicates the Hop1 stained nucleolus in *pch2Δ*. (P) Numbers of Zip3 foci per nucleus determined in spread nuclei in *dmc1Δ* (t = 5 hrs), WT (t = 7 hrs), *pch2Δ* (t = 7 hrs), *PCH2ndt80Δ* (t = 8 hrs), *pch2Δndt80Δ* (t = 8 hrs), respectively. P-values are from two-sided Wilcoxon rank sum tests. Error bars represent 95% confidence intervals. (See also Figure S2 for analysis of Hop1 domains in WT and *pch2Δ*.) (Q–V) Nuclei from WT (Q–S) or *pch2Δ* (T–V) from parallel time courses were spread and stained with antibodies against Hop1 and Zip1. Note discontinuous staining of Hop1 and Zip1 in WT (Q,R) compared to continuous staining patterns for both proteins in *pch2Δ* (T,U). (W) Twenty well-spread nuclei were selected and the combined contour length of Zip1 and Hop1 was determined in WT and *pch2Δ* (see Materials and Methods). The average SC/axis length was 34 µm (±1.8 µm C.I.) in WT and 40 µm (±2.2 µm C.I.) in *pch2Δ*, indicating a significant increase in *pch2Δ* versus WT (p = 0.00023; two-sided Wilcoxon rank sum test). Error bars represent 95% confidence intervals. Meiosis was induced at 33°C.

In *dmc1Δ* at 33°C, Zip3 localizes to nuclei abundantly, although at reduced numbers. At a time when most cells have completed DSB formation (t = 5 hrs; G.V.B., unpublished data), 33 (±7 S.D.) Zip3 foci are detected (n = 58 nuclei), compared to ∼50 Zip3 foci in WT cells (Figure 4A–4C and 4P). In *dmc1Δ*, maximum Zip3 localization is reached at t = 5 hrs, as indicated by comparable numbers of foci at t = 4 and t = 6 hrs (data not shown). Thus, Dmc1 is required for association of Zip3 with meiotic chromosomes at normal levels. Hop1 also localizes at high levels to meiotic chromosomes in *dmc1Δ*, but poor spreading in these cells interferes with quantitation of Hop1 foci. Of the Zip3 foci detected in *dmc1Δ*, 77% (±11% S.D.) colocalize with Hop1, compared to 26% (±8% S.D.) fortuitous colocalization. About 10% of *dmc1Δ* nuclei further exhibit WT-like patterns of Zip3 staining, with several Zip3 foci located in a linear array, consistent with staining along condensed chromosome axes. In these nuclei, Zip3 again colocalizes with-Hop1 at high levels (Figure 4A–4C). In summary, Zip3 foci form with reduced numbers in *dmc1Δ*, but tend to be associated with Hop1.

In *ndt80Δ* at 33°C, at a time when most cells have undergone pachytene arrest (t = 8 hrs), Zip3 and Hop1 localize to meiotic chromosomes with patterns and numbers similar to wild-type pachytene nuclei (compare Figure 4J and 4K with Figure 4D and 4E). Both Zip3 and Hop1 are detected as foci, with 50 (±9 S.D.) Zip3 foci and 63 (±8 S.D.) Hop1 foci detected (n = 51 nuclei). Colocalization between Hop1 and Zip3 is also high in *ndt80Δ*, with 76% (±11% S.D.) of Zip3 foci colocalizing with Hop1, similar to the WT pachytene stage (n = 51 nuclei; Figure 4F and 4L).

We conclude that Dmc1 is required for normal levels of both Zip3 localization and Hop1-Zip3 co-staining domains. Importantly, association of Zip3 and Hop1 is independent of *NDT80*, indicating that it is established prior to and independent of double Holliday junction resolution into COs.

### Pch2 controls levels and distribution of Hop1 loading

Pch2's role in chromosome morphogenesis was examined in more detail by analyzing patterns and levels of Hop1 localization in *ndt80Δ*-arrested cells. Detection of 63 (±8 S.D.) Hop1 foci in *ndt80Δ* confirms that Hop1 localizes as foci rather than in lines along pachytene-arrested chromosomes (Figure 4K). Conversely, in both *NDT80* and *ndt80Δ* nuclei, with maximized visualization of near-background signals, Hop1 foci frequently coalesce into lines, consistent with continuous localization of Hop1 at base levels along pachytene chromosomes (data not shown).

Absence of Pch2 affects Hop1 patterns similarly in *ndt80Δ* and *NDT80* (compare Figure 4H and 4N; ref. [37]): Hop1 localizes as continuous, mostly uniform lines along the 16 homolog pairs. Quantitative analysis further identifies roles of Pch2 in controlling both Hop1 loading levels and patterns: Hop1 signal intensities are ∼three-fold increased in *pch2Δ* (p<0.0001; see Figure S2A for details). The Hop1 staining observed in *pch2Δ* could be due to a uniform increase exclusively or concurrent Hop1 redistribution. To examine this question, number and contour length of high intensity Hop1 signals were determined in 20 WT and *pch2Δ* nuclei (see Materials and Methods for details). In *pch2Δ*, strong Hop1 signals exhibit ∼two-fold increased average contour lengths and are present at reduced numbers (see Figure S2B, S2C; p<0.0001; two-sided Wilcoxon rank sum test). If extra loading had occurred universally, patterns of strong Hop1 signals would be similar in WT and *pch2Δ*. We conclude that the more uniform Hop1 signal in *pch2Δ* is due to an overall increase in Hop1 signal intensities concurrent with changes in Hop1 loading patterns.

Together, these findings have four important implications. (i) Hop1 is a prominent component of pachytene SC. (ii) In WT, Hop1 is present along chromosome axes in a mostly continuous pattern at base levels, with hyperabundance at distinct chromosome domains [37]. (iii) Pch2 controls both overall levels and patterns of Hop1 localization along meiotic chromosomes. (iv) Changes in Hop1 localization are caused by absence of Pch2, rather than being an indirect result of meiotic arrest.

### Pch2 controls axis morphogenesis and number of crossover-designated sites

Next, the role of Pch2 in controlling Zip3 association with chromosomes was investigated in WT and *pch2Δ* at a time point exhibiting maximum levels of pachytene cells (>50%; t = 7 hrs) as well as in *ndt80Δ*-arrested cells (T = 33°C): In WT, Zip3 and Hop1 predominantly localize as distinct foci (Figure 4D–4F; see above). In *pch2Δ* (t = 7 hrs), by contrast, Hop1 localizes in continuous lines and Zip3 foci are occasionally not well separated (Figure 4G; see above; ref. [37]). Further, in *pch2Δ*, Hop1 (but not Zip3) is detected in the nucleolus (Figure 4H) [33].

In WT nuclei, 56 (±1.7 S.E.) Zip3 foci are detected along meiotic chromosomes (n = 68, see above), while in *pch2Δ*, the average number of Zip3 foci per nucleus is 62 (±1.5 S.E.) (n = 64 nuclei; Figure 4D, 4G, and 4P). Accordingly, the number of Zip3 foci in *pch2Δ* is significantly increased (P = 0.0026, two-sided Wilcoxon rank sum test). To exclude possible effects of differences in meiotic progression, the number of Zip3 foci in WT and *pch2Δ* was also examined in the *ndt80Δ* background. In *PCH2ndt80Δ*, 50 (±1.3 S.E.) Zip3 foci are detected, compared to 67 (±1.3 S.E.) Zip3 foci *pch2Δndt80Δ*, reflecting an increase by 34% (Figure 4P). Again, this increase is statistically significant (p<0.0001, two-sided Wilcoxon rank sum test). Thus, Pch2 controls the number of Zip3 foci along pachytene chromosomes. Notably, increased numbers of Zip3 foci are not caused by accumulation of cells at the pachytene stage in *pch2Δ*: In *ndt80Δ* arrested cells, the number of Zip3 foci is substantially increased in *pch2Δ* compared to the corresponding *PCH2* strain, indicating that Pch2 controls the number of Zip3 association sites.

To examine effects of *pch2Δ* on chromosome axis length, SC contour length was measured by staining for Hop1 and Zip1. In WT pachytene nuclei (identified based on Zip1 staining), Hop1 and Zip1 preferentially localize to alternating domains, whereas largely overlapping localization patterns are observed in *pch2Δ* pachytene cells (Figure 4S and 4V) [37]. The combined Hop1/Zip1 SC contour length of an entire chromosome complement is 34 µm (±0.9 µm S.E.) in WT, in accordance with published results (see ref. [41]). In *pch2Δ*, the axis length is increased by 18% to 40 µm (±1.2 µm S.E.), representing a significant increase (p = 0.00023, two-sided Wilcoxon rank sum test; see Figure 4W). Thus, homolog axes fail to shorten appropriately in the absence of Pch2.

Roles of Pch2 in controlling the number of Zip3-marked presumed CO-designated sites, Hop1's localization to the same regions, and meiotic chromosome axis length as well as Pch2's role in CO interference have important implications for the mechanism of CO control (Discussion).

### Double crossovers contribute disproportionately to genetic distances in *pch2Δ*

**Figure 5:**
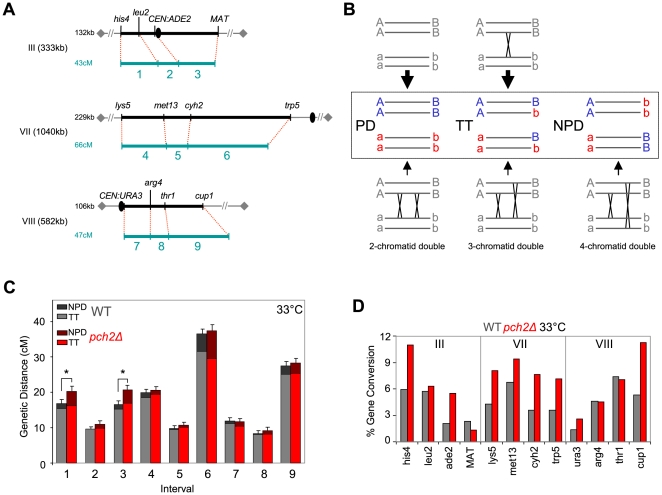
Genetic map distances in WT and *pch2Δ* determined by tetrad analysis at 33°C. (A) Test intervals on chromosomes III, VII, and VIII. Marker order, physical (kb) and genetic sizes (cM) of surveyed chromosome regions are shown. Ovals indicate centromeres; diamonds represent telomeres. Intervals are referred to by numbers 1–9 throughout the text. (B) Classes of tetrads for a given interval and contributing crossover (CO) events. Parental ditype tetrads (PD), generated in the absence of COs, and tetratype tetrads (TT), generated when a single CO occurs, are predominant tetrad classes. Nonparental ditype tetrads (NPD) are generated exclusively by double COs involving all four chromatids. Double COs involving three chromatids produce TTs, and those involving two chromatids produce PDs. (C) Genetic distances determined for intervals 1–9 (see A) at 33°C. Contributions of TTs and NPDs to map distances are indicated in different shades. Asterisks indicate significant differences between map distances in WT and *pch2Δ* strains. Error bars represent standard errors (see also Table 1). (D) Frequencies of non-Mendelian segregation ( = gene conversion) events (i.e. markers deviating from 2∶2 segregation) in WT and *pch2Δ* at 33°C.

We examined the roles of Pch2 in recombination in an interference tester strain carrying twelve pairs of heterozygous markers, defining nine genetic intervals along three homologs (designated as intervals 1 to 9 in Figure 5A), [42]. Chromosomes III, VII, and VIII represent small, large and intermediately sized yeast chromosomes, respectively. Marked regions span physical distances of 132 kb, 229 kb and 106 kb, corresponding to WT map distances of 43 cM, 66 cM and 47 cM, respectively (Figure 5A; below).

Map distances were determined using tetrads with four viable spores and Mendelian (i.e. 2∶2) segregation at a given pair of markers: Three types of tetrads can be distinguished (Figure 5B, boxed region): (i) All four spores exhibit parental marker combinations, giving rise to a parental ditype (PD); (ii) two spores are parental and two recombinant, constituting a tetratype (TT); (iii) all four spores carry nonparental marker configurations, constituting a nonparental ditype (NPD). The majority of PDs are derived from tetrads where no CO has occurred. TTs preferentially arise from tetrads that have undergone a single CO, while NPDs are derived from double COs involving all four chromatids within an interval (Figure 5B).

Double COs involving two or three chromatids give rise to PDs or TTs, respectively, and are indistinguishable from tetrads involving a single or no CO (Figure 5B, lower part). Thus, total frequencies of double COs are extrapolated from NPD frequencies [43]. Note that this formula assumes absence of chromatid interference which has been validated for WT and *pch2Δ* (data not shown).

Following meiosis at 33°C, tetrads from WT and *pch2Δ* strains were dissected and markers were scored. Dissection of WT and *pch2Δ* asci gave rise to >1200 four spore-viable tetrads for each strain. In WT, genetic distances are similar to those previously reported (Figure 5C; Table 1), [42]. Map distances are remarkably similar between WT and *pch2Δ*, with two of nine intervals in *pch2Δ* exhibiting a significant increase (intervals 1 and 3). We note an apparent increase in NPD frequencies in *pch2Δ*. Accordingly, in *pch2Δ* double COs when calculated separately, contribute disproportionally to total map distances in seven intervals (see Figure 5C, no differences in intervals 4 and 9).

**Table 1:** Effects of *pch2Δ* on genetic distances and crossover interference in intervals along three chromosomes at 33°C.

Genotype		Chromosome III			Chromosome VII			Chromosome VIII		
		his4-leu2	leu2-CEN3	CEN3-MAT	lys5-met13	met13-cyh2	cyh2-trp5	CEN8-arg4	arg4-thr1	thr1-cup1
Interval name		(1)	(2)	(3)	(4)	(5)	(6)	(7)	(8)	(9)
Interval size	kb	25	21	86	57	36	136	35	19	52
Wild type	P∶N∶T	717∶5∶319	872∶0∶207	761∶5∶333	663∶5∶388	863∶1∶201	396∶20∶673	852∶3∶242	870∶1∶166	507∶9∶508
	cM	16.8±0.9	9.6±0.6	16.5±0.9	19.8±0.1	9.7±0.7	36.4±1.3	11.9±0.8	8.3±0.6	27.4±1.1
	P*	.010	.017	.008	<.0001	.059	<.0001	.090	.163	<.0001
pch2Δ/”	P∶N∶T	688∶15∶328	875∶4∶216	743∶15∶381	633∶4∶402	829∶2∶211	425∶31∶616	910∶5∶240	925∶4∶179	499∶11∶513
	cM	20.3±1.3	11.0±0.8	20.7±1.2	20.5±0.9	10.7±0.7	37.4±1.6	11.7±0.8	9.2±0.8	28.3±1.2
	P*	.667	.400	.212	<.0001	.098	<.0001	.416	.974	<.0001

Map distances and standard errors (in centiMorgans; cM) were calculated from parental ditypes (PD), nonparental ditypes (NPD) and tetratypes (TT) according to Materials and Methods.

*: P-values <0.05 indicate interference. P values indicate the probability that deviations of the observed PD∶NPD∶TT distribution from the distribution expected for no interference are due to chance [44].

Thus, Pch2 is not required for formation of COs at normal levels in most genome regions, consistent with prior physical analysis at a particular recombination hotspot [35],[37]. However, Pch2 appears to limit the occurrence of closely spaced double COs.

### Pch2 mediates normal levels of crossover interference

**Figure 6:**
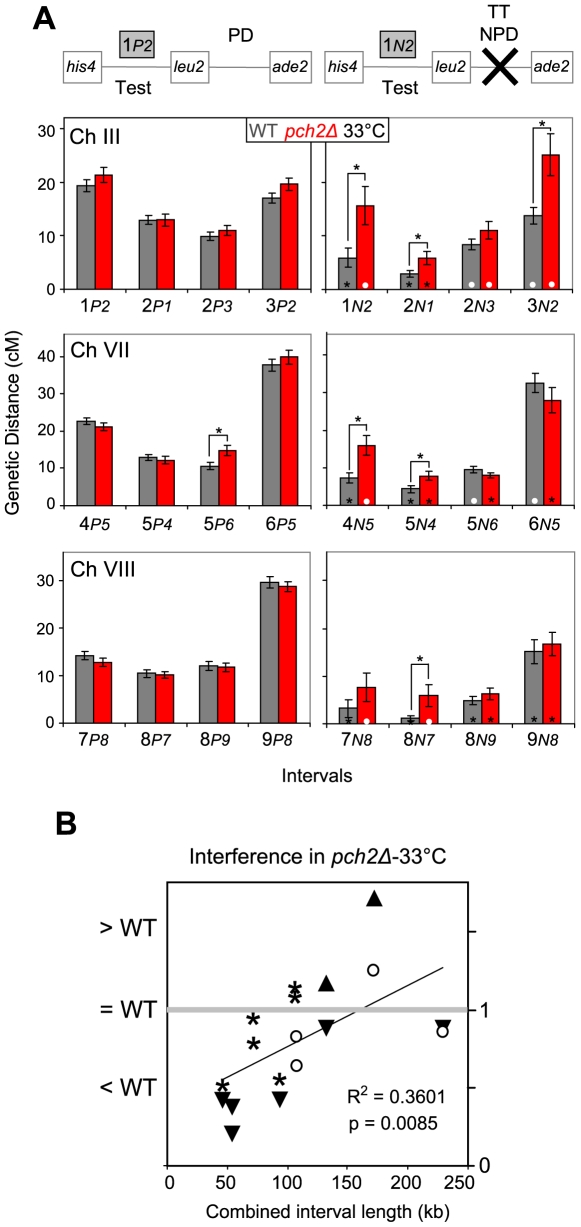
Interference in WT and *pch2Δ* in nine intervals at 33°C. (A) Modified coincidence analysis. Left column: Map distances for test intervals ( = Test) determined from tetrads with parental reference intervals (PD). Right column: Map distances for test intervals determined from tetrads with nonparental reference intervals (TT, NPD). Interval names are given as follows: Test intervals are indicated by the first number in bold, CO status of the reference interval is indicated by the letter in italic (*P* = parental; *N* = nonparental), and reference intervals are specified by the number in italic. For interval numbers see Figure 5A. Error bars are standard errors. Asterisks above bars indicate significant differences for map distances in bracketed intervals between genotypes. Asterisks on bars in right column indicate significant differences between map distances for test intervals within genotypes (indicating interference). White dots on bars in right column indicate lack of significant differences between the same pairs of map distances within a genotype (indicating absence of interference). (B) Strength of CO interference as a function of physical interval size. Combined interval length (kb) indicates the physical distance between the two outermost markers of intervals considered. Ratios of map distances (P/N) were determined and compared between WT and *pch2Δ*. (Ratios <1 indicate loss of interference in *pch2Δ* and ratios >1 indicate increased strength of interference in *pch2Δ* versus WT). Symbols: *, significant interference in WT and *pch2Δ*; ○, no significant interference in WT and *pch2Δ*; ▾, significant interference in WT, but not in *pch2Δ*; ▴, significant interference in *pch2Δ*, but not in WT.

Increased levels of double COs in *pch2Δ* raise the question of Pch2's role in CO control. Modified coincidence analysis and analysis of NPD frequencies were used to determine effects of *pch2Δ* on CO interference using the tetrad set generated at 33°C. In modified coincidence analysis, map distances for each test interval are determined for two distinct tetrad subsets [31]: Subset P includes tetrads with parental marker configuration at an adjacent reference interval (PD; Figure 6A, left column). Subset N includes tetrads with non-parental marker configuration at the reference interval (TT or NPD; Figure 6A, right column; Table S1).

Map distances derived from subset P are remarkably similar between WT and *pch2Δ*, with only a single interval exhibiting a significant increase in *pch2Δ* CO frequencies (interval 5*P6*; Figure 6A, left panel). In contrast, map distances derived from subset N are strikingly different between WT and *pch2Δ* (Figure 6A, right panel): In six out of 12 adjacent interval pairs, map distances are significantly increased in *pch2Δ* compared to WT. Thus, Pch2 has no detectable effect on CO frequencies along an interval when the adjacent interval is parental, but suppresses CO formation in the same interval when the adjacent interval is recombinant. Notably, total map distance increases in intervals 1 and 3 in *pch2Δ* can entirely be attributed to subset N tetrads, while map distances in subset P tetrads are indistinguishable between WT and *pch2Δ* (Figure 5C; Figure 6A, left panel).

#### Modified coincidence analysis

Interference for each interval pair was examined by comparing map distances derived from subset P and subset N tetrads. A reduced map distance in subset N versus subset P tetrads is indicative of interference. As an example, in WT, the map distance in test interval 1 is 19 cM when determined from subset P tetrads ( = 1*P2*, left panel Figure 6A, grey bar), but 7 cM for the same test interval when determined from subset N tetrads ( = 1*N2*, right panel Figure 6A, grey bar). Thus, interval 2 strongly interferes with COs in interval 1. (Note that center intervals are measured twice).

In WT, eight reference intervals interfere significantly with COs in adjacent test intervals (Figure 6A; asterisks on bars), while no interference is detected between the remaining four interval pairs (white dots). In *pch2Δ*, interference is abolished in four interval pairs that exhibit interference in WT. Moreover, interference is reduced in one interval pair (see Table S1). Interference remains intact between interval pairs 8 and 9. An apparent gain of interference is observed between interval pairs 5 and 6 (see Figure 6A, discussion).

The strength of interference between two intervals can be inferred from the ratio of map distances in subset N over subset P tetrads ( = N/P), with N/P ratios substantially <1 indicating strong interference. In WT at 33°C, N/P ratios were lowest (i.e. <0.5) for interval pairs spanning <100 kb, consistent with interference operating over ∼100 kb (Table S1). In *pch2Δ*, N/P ratios were generally increased compared to WT, most strikingly between 50–100 kb. A direct comparison of N/P ratios between WT and *pch2Δ* at various physical distances further confirms that interference in *pch2Δ* tends to be reduced most dramatically at physical distances below 100 kb, while it is equally strong as WT interference or stronger >100 kb (Figure 6B). These findings indicate that interference in *pch2Δ* is reduced or abolished over short physical distances, but is robust over longer distances, possibly revealing a long range interference system active in the *pch2Δ* mutant (see Discussion).

#### Analysis of non-parental ditype frequencies

Effects of *pch2Δ* on CO interference were further examined by determining frequencies of NPDs. Without interference, NPDs occur at frequencies inferred from TT frequencies, while the presence of interference results in a significant decrease of NPDs [44]. In WT, six out of nine intervals exhibit significant interference (Table 1). In *pch2Δ*, interference is lost in all three intervals along chromosome III, and is reduced in intervals 7 and 8 [41]. Conversely, *pch2Δ* exhibits WT-like levels of interference in intervals 4, 6 and 9. (NPD ratios give similar results, but were not used due to caveats of this analysis [44]). Notably, interference as assayed by NPD frequencies is again preferentially lost or reduced in *pch2Δ* in intervals <100 kb (Table 1).

Together, three conclusions emerge from this analysis: (i) Pch2 is dispensable for CO formation in intervals flanked by parental genome regions. By implication, major CO pathways function normally in the absence of Pch2. (ii) Conversely, Pch2 is required specifically for reducing COs in response to COs in adjacent chromosome regions. (iii) Pch2 is preferentially required for CO interference in intervals ranging from 20 kb to 100 kb. CO interference across intervals appears not to depend on Pch2 (Discussion).

### Spore viability and crossover distribution indicate normal crossover assurance in *pch2Δ*

**Figure 7:**
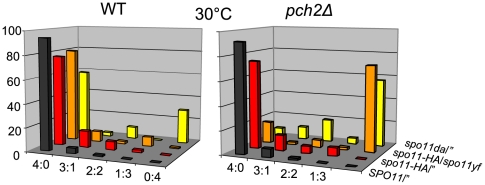
Spore viability patterns at reduced DSB levels in WT and *pch2Δ* at 30°C. Tetrads were dissected from strains with the indicated genotypes of *spo11* alleles. Ratios 4∶0, 3∶1, 2∶2, etc. indicate the frequencies of four-spore viable, three-spore viable, two-spore viable, etc. tetrads. *spo11* hypomorphic mutants form ∼80% (*spo11-HA/”*), ∼30% (*spo11-HA/spo11yf*), and ∼20% (*spo11da/”*) of WT DSB levels [29].

**Figure 8:**
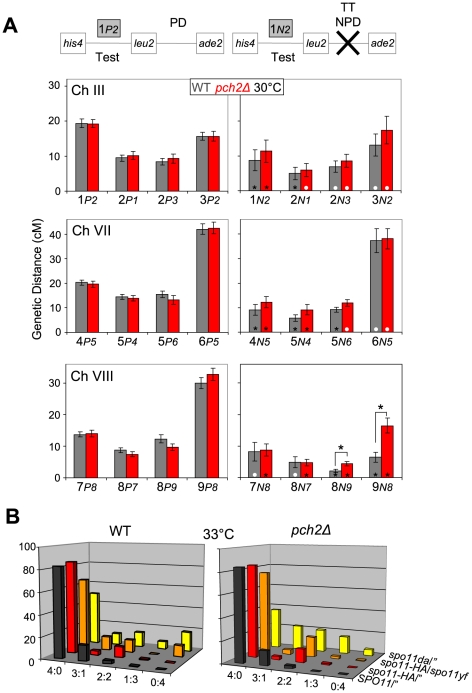
CO interference and spore viability in *pch2Δ* and WT at different temperatures. (A) Modified coincidence analysis for the same strains shown in Figure 6, sporulated at 30°C. Note that the apparent lack of interference between intervals 7 and 8 is likely due to large standard errors in the relatively smaller data set (see Table S1). See Figure 6 legend for details. (B) Spore viability patterns indicative of homolog nondisjunction for the same strains analyzed in Figure 7, sporulated at 33°C. See Figure 7 legend for details.

Numerous mutants defective for CO interference also exhibit intermediate to severe defects in CO assurance, as suggested by frequent occurrence of tetrads with two viable or zero viable spores due to homolog nondisjunction (e.g. [28],[32]). Such patterns of spore viability are frequently associated with elevated levels of non-exchange chromosomes [32]. WT-like patterns of spore viability in *pch2Δ* provide no indication of increased homolog nondisjunction: Overall spore viability in *pch2Δ* at 33°C is 84.0% compared to WT viability of 82.6%, consistent with normal homolog disjunction in *pch2Δ*. WT like levels of spore viability are also observed in *pch2Δ* at 30°C (see Figure 7 and Figure 8B: *SPO11/”*, black bars; [33]).

Low levels of non-exchange homolog pairs could be rescued by a backup system that mediates disjunction of non-exchange chromosomes reducing the reliability of spore viability as a measure for CO assurance [45]. To directly evaluate whether chromosomes receive similar numbers of COs in WT and *pch2Δ*, tetrads formed at 33°C were therefore individually inspected for the number of COs along three pairs of homologs. Tetrads with no CO in the monitored interval are only marginally increased in *pch2Δ*, by 1%, 6% and 8%, suggesting that similar numbers of COs are formed along a given interval in WT and *pch2Δ* (Figure S3). Taken together, patterns of spore viability and WT-like levels of COs across the chromosome segments examined suggest that CO assurance is functional in *pch2Δ*. These findings raise the possibility that CO assurance and CO interference can be separated (discussion).

### Increased gene conversions are equally associated with crossovers and noncrossovers in *pch2Δ*

Non-Mendelian marker segregation during meiosis (e.g., 3∶1 or 1∶3) occurs due to gene conversion of markers in association with meiotic recombination [31]. Gene conversion frequencies are increased in *pch2Δ* at eight markers, 1.2- to 2.0 fold over WT (Figure 5D; Table S2). Thus, Pch2 plays a role in suppressing gene conversion. A gene conversion may be flanked by parental or recombined chromosome arms, suggesting association with a NCO or a CO, respectively. In WT, at the assayable six central markers, gene conversions are associated with COs and NCOs at similar frequencies. In *pch2Δ*, at markers where gene conversion is substantially increased (*ade2*, *met13*, *cyh2*), such events are also flanked by COs and NCOs with similar frequencies (Figure S4). Thus, *pch2Δ* increases occurrence of gene conversions in both CO and NCO interactions. Increased gene conversion could be due to changes in the length of heteroduplex in recombination intermediates, repair defects, and/or region-specific changes in DSB levels (see discussion). Notably, *pch2Δ* does not affect DSB levels at a hotspot of recombination and does not change global DSB patterns along the majority chromosomal loci (A. Hoachwagen, personal communication; [35],[37]).

### Suppression of *pch2Δ* crossover interference defects at lower temperatures

Meiotic phenotypes in several mutants are dramatically modulated by incubation temperature, with prominent effects on processing of recombination intermediates and formation of CO products (e.g., [11],[37]). In *pch2Δ*, temperature modulates defects in recombination progression, but not those in chromosome domain organization [37]. To examine whether the interference defect observed in *pch2Δ* at 33°C is affected by temperature, we investigated crossover formation and interference also at 30°C. Surprisingly, this minor temperature change results in a drastic improvement in CO interference in *pch2Δ*. At 30°C, map distances along three chromosomes are similar between *pch2Δ* and WT (i) for total tetrads, without increases in NPDs (Figure S5) and (ii) for subset P tetrads (Figure 8A, left panel). Also, and in sharp contrast to observations at 33°C, map distances in subset N tetrads exhibit only minor differences between *pch2Δ* versus WT. Only interval pairs 8*N9* and 9*N8* exhibit significantly higher CO frequencies in *pch2Δ* (Figure 8A, right panel). Modified coincidence analysis suggests that in *pch2Δ* at 30°C, interference is lost in only two interval pairs (Figure 8A; interval pairs 2*-1* and 5-*6*). NPD frequencies further indicate loss of interference in *pch2Δ* at 30°C in only one interval (Table S4). Thus, defects in crossover interference can be suppressed by incubation at lower temperatures.

### Pch2 ensures formation of viable spores when DSBs are reduced

The role of Pch2 in meiosis when DSBs are limiting was examined in hypomorphic *spo11* strain backgrounds. In WT meiosis, normal homolog segregation is maintained despite reduction of initiating DSBs to ∼20%, of normal levels, likely due to preferential processing of DSBs into COs versus NCOs [29]. Levels of initiating DSBs are reduced to ∼80%, ∼30% or ∼20% of normal levels in strains homozygous for *spo11*-*HA*, heterozygous for alleles *spo11yf*-*HA* ( = *spo11yf*) and *spo11*-*HA* or homozygous for *spo11da*-*HA* ( = *spo11da*), respectively [29].

Patterns of tetrad viability in *PCH2* and *pch2Δ* strains were determined following sporulation at 30°C on solid medium (n≥97 tetrads). Frequencies of four spore-viable tetrads in WT indicate normal chromosome segregation in >58% of cells despite DSB reduction to ∼20% of WT levels consistent with earlier findings (Figure 7; Table S3) [29]. Frequency of four spore-viable tetrads decreases dramatically in *pch2Δ* strains hypomorphic for *spo11*, in particular when DSBs occur are reduced below levels occurring in a homozygous *spo11*-*HA* strain. Chromosome segregation occurs normally in only 18% and 8% of meioses in *spo11yf*/*spo11*-*HA* and homozygous *spo11da/”* strains, respectively, and >50% of meioses in the same strains generate zero spore-viable tetrads. Such viability patterns can occur when≥two homolog pairs missegregate. We conclude that Pch2 plays a critical role for spore viability when DSBs are reduced. Thus, although Pch2 does not play a role in spore viability at normal DSB levels, it is essential under conditions of reduced DSB formation.

### Incubation conditions modulate spore viability in *pch2Δ* at reduced DSB levels

Following observation of temperature-modulated interference in *pch2Δ*, we next examined whether incubation conditions also affect spore viability in *pch2Δ* at reduced DSB levels. Examining effects of hypomorphic *spo11* on spore viability at 33°C on solid medium, we find, surprisingly, that spore viabilities are high in the *pch2Δ* strain, a drastic deviation from observations at 30°C (compare Figure 7 and Figure 8B). Notably, in *spo11yf/spo11*-*HApch2Δ* at 33°C, 75% of tetrads undergo normal meiotic chromosome segregation as suggested by levels of 4 spore-viable tetrads, compared to 18% 4 viable spore tetrads in the same strain sporulated at 30°C in parallel (see Figure 7, orange bars). Similar results are obtained for *pch2Δ* strains homozygous for *spo11da/”* (compare Figure 7 and Figure 8, yellow bars) or for *spo11da*/*spo11yf* (data not shown): At 33°C, these strains give rise to 37% and 81% four spore-viable tetrads, compared to frequencies of 8% and 2%, respectively, at 30°C. (In *spo11daPCH2*/*spo11yfPCH2* ∼48% of tetrads give rise to four viable spores at both 33°C and 30°C). In subsequent experiments, we also discovered that spore viability patterns in *pch2Δ* strains hypomorphic for *spo11* are also affected by culture conditions (see below).

In summary, higher versus lower temperatures oppositely modulate *pch2Δ* defects in CO interference and spore viability at reduced DSB levels. Conditions that improve spore viability weaken or eliminate interference and vice versa. Together, these results have several implications: (i) CO interference and factors affecting spore viability at reduced DSB levels can be uncoupled in *pch2Δ*. (ii) Effects of temperature on CO interference and the process that mediates normal spore viability at reduced DSB levels suggest linkage via Pch2 between both processes. (iii) Pch2 stabilizes both CO interference and spore viability over a wide range of DSB levels, temperatures, and possibly other environmental conditions (see below).

### Reduced spore viability in *pch2Δ* despite normal crossover levels at a recombination hotspot

**Figure 9:**
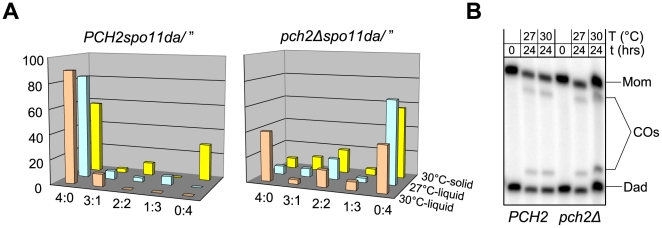
Spore viability and crossovers in *pch2Δ* under different incubation conditions. (A) Spore viability patterns in *PCH2spo11da/”* (left) and *pch2Δspo11da/”* (right), undergoing meiosis at 30°C (liquid medium), 27°C (liquid medium), or 30°C (solid medium). At least 30 tetrads were dissected under each condition. (B) Crossover levels at the *HIS4LEU2* recombination hotspot. Meiotic cultures were split at t = 0 hrs, and crossover levels were determined after incubation at 30°C or 27°C for 24 hrs. Asci from the same cultures were dissected to determine effects on spore viability (see A).

Additional effects of incubation conditions in *pch2Δ* were revealed during our investigation of recombination defects at reduced DSB levels. Physical recombination analysis is routinely performed in liquid medium, while spore viability is determined following sporulation on solid medium. To ascertain correspondence between these conditions, asci from parallel cultures incubated at 30°C with solid or liquid medium were dissected and viability patterns were compared. Surprisingly, WT and *pch2Δ* strains carrying *spo11-da*/” formed four viable spore tetrads at much higher levels when sporulated at 30°C in liquid versus solid medium (Figure 9A, compare pink and yellow bars).

To examine CO formation in *pch2Δspo11da*/” under conditions that result in low levels of four viable spore tetrads, sporulation in liquid medium was analyzed at 27°C and 30°C: In *pch2Δspo11da*/”, incubation of parallel cultures results in a dramatic decrease in spore viability at 27°C versus 30°C, while viability patterns are similar under both conditions in *PCH2* (Figure 9A, pink and blue bars). Accordingly, in *pch2Δ* hypomorphic for *spo11*, spore viability is modulated not only by temperature, but also by the exact nature of the sporulation medium.

Final CO levels were examined in *PCH2* and *pch2Δ* in a *spo11da/”* background at the *HIS4LEU2* hotspot of recombination (see ref. [11] for details). Surprisingly, CO levels in *PCH2* and *pch2Δ* were extremely similar; both at 27°C and 30°C, in four independent WT and *pch2Δ* strains (Figure 9B and data not shown). Thus, differences in CO formation, at least at the *HIS4LEU2* recombination hotspot, are not responsible for the loss in spore viability in *pch2Δ* at reduced DSB levels.

In summary, *pch2Δ* is defective in ensuring normal spore viability when overall DSB levels are reduced, with viability patterns suggestive of homolog disjunction defects. *pch2Δ* may affect genome-wide levels or distribution of initiating DSBs, the efficient designation of DSBs as future COs in genomic regions outside of the *HIS4LEU2* hotspot or the formation of functional chiasmata (see Discussion). Importantly, our results identify Pch2 as a protein that ensures normal homolog segregation at reduced DSB levels. Reduction of DSBs or absence of Pch2 alone have marginal or no effects on homolog segregation, yet both mutant conditions combined synergistically affect spore viability.

## Discussion

The present work provides novel insights into the question of how chiasma distribution is controlled along the genome of sexually reproducing organisms. We demonstrate a spatial association between CO-designated sites and structurally differentiated chromosome axes: Early during meiotic prophase, axis proteins Hop1 and Red1 preferentially associate with sites designated to become COs. A key implication of this finding is that local modifications of chromosome axis and recombination site selection are coordinated, and are possibly controlled by the same determinants. Pch2 controls the association of Hop1 with designated CO sites, the number of designated CO sites along the genome and CO interference. Identification of *pch2Δ* as a mutant that affects CO/chiasma formation in response to remote recombination events without playing a major role in overall CO levels suggests that CO control is mechanistically distinct from and likely superimposed on basic recombination events. Based on these data, we propose a model of CO control in which meiotic chromosomes become organized into multiple modules of assured CO formation, with concurrent imposition of crossover interference. Pch2 is proposed to function as a size determinant for these modules of chiasma assurance and interference.

### Correspondence between Hop1-enriched axis domains and crossover designated sites

Pachytene chromosomes in yeast display a domainal organization, where Hop1/Red1 or Zip1-enriched regions occur in an alternating pattern [37]. Our analysis supports the idea that hyperabundance domains are layered over a base level of Hop1 (and Zip1) along the lengths of synaptonemal complexes. Correspondence between Hop1 hyperabundance domains and CO-designation marker Zip3 establishes a link between domainally modified chromosome axes and positions of future COs [21]–[23]. Crossovers and CO designation markers Zip2/Zip3 occur at different sites in each cell and exhibit interference distribution [22],[46]. By implication, Hop1 hyperabundance domains likely also form at different positions in different cells along a given chromosome. Together, these findings suggest that chromosome axes undergo a differentiation process that is spatially coordinated with crossover placement.

During WT meiosis, association between Zip3 and Hop1 reaches maximum levels in pachytene nuclei. High levels of Zip3-Hop1-association are also detected in *ndt80Δ* cells arrested at the pachytene stage (Figure 4L), indicating that association is established prior to and independent of double Holliday junction resolution, a recombination step blocked in *ndt80Δ* [10]. Thus, Hop1-Zip3 association is completed prior to and independent of completion of the majority of COs.

Our findings further suggest that association between Hop1/Red1 and Zip3 is established during the zygotene stage and can occur independently of stable strand invasion. Zip3 is the earliest known marker for designated CO sites [22]. In pre-zygotene cells, Zip3 localizes to paired centromeres of yeast chromosomes [24]. During the zygotene stage, Zip3 appears to localize abundantly to additional sites, a process that is completed at the pachytene stage when Zip3 is found at interference-distributed CO designation sites, while it is absent from centromeres [24]. In the population of zygotene nuclei analyzed here, ∼50 Zip3 foci are detected, suggesting that Zip3 occupies multiple non-centromeric positions at this stage. Zip3 foci colocalize at high levels with Red1 in the same zygotene cells (Figure 3I–3M). Thus, association between Red1/Hop1 and Zip3 at designated CO sites appears to be established during the zygotene stage.

In *dmc1Δ*, a mutant defective for strand invasion, Zip3 localizes to chromosomes with numbers substantially lower than those observed in normal zygotene nuclei. A subset of these cells, however, exhibit Zip3 localization with WT-like numbers and patterns (Figure 4C). A high percentage of Zip3 foci is associated with Hop1 in such nuclei, raising the possibility that Zip3-Hop1 association can occur independent of Dmc1-mediated strand invasion. Whether Zip3 localizes to its normal sites in *dmc1Δ* cells is presently unknown.

Hop1/Red1 localize to meiotic chromosomes prior to and independent of DSB formation, consistent with functions at earlier stages (G.V.B., unpublished; [13],[17]). Several scenarios can explain the transition between early, pre-DSB association of Red1/Hop1 with meiotic chromosomes and their association with Zip3 marked designated CO sites at later stages: Red1/Hop1 may (i) become associated with future CO sites as an outcome of CO designation, following relocalization from a more dispersed (pre-)leptotene localization pattern; (ii) initially be present at all nascent recombination interactions and later undergo selective stabilization at future CO sites; (iii) preferentially localizes to future CO sites prior to CO designation, possibly participating in CO designation itself. (iv) Finally, it is possible that Zip3 preferentially localizes to Hop1/Red1 hyperabundance domains due to preferential recombination initiation in such domains [17]. Further work is required to determine whether Hop1/Red1 hyperabundance domains assemble at CO designated chromosomal positions before or after CO designation, and whether CO designation is a requirement for association between Hop1 and Zip3.

Spatial association between structural axis modifications and markers of nascent recombination interactions have also been observed in other organisms. (i) In *Sordaria*, cohesin associated protein Spo76/Pds5 is depleted from Msh4-marked recombination sites in a mutant deficient for the meiotic cohesin Rec8. Local splitting of sister chromatids at the corresponding sites also occurs in the WT, and it was proposed that intersister connections may become destabilized as part of the normal process of chiasma formation [16]. (ii) In an *ATM^−/−^* mouse, SC proteins Sycp3 and Sycp1 are absent from sites of ongoing recombination [47]. (iii) In *C. elegans*, SC component SYP-1 and axis proteins HTP-1/2 are removed from reciprocal chromosome arms directed by and dependent on designation of a given recombination interaction as future CO [48]. These data indicate that locally weakened sister cohesion and enhanced interhomolog interactions may both contribute to preferential interhomolog recombination. Such modifications may be especially important for CO formation which entails long-lived strand invasion intermediates [10],[11]. In yeast, axis ensemble Hop1/Red1 plays a central role in directing meiotically induced DSB processing towards homologous chromosomes, with Mec1-dependent Hop1 phosphorylation constituting a key event in establishing this interhomolog bias [19].

Our results further provide insights into likely dynamics of SC assembly. SC initiation occurs at Zip3 foci, some of which correspond to CO-designated sites [21] (N.J., unpublished data). Conversely, domains enriched for Zip1 alternate with Hop1/Red1 (and Zip3) enriched domains when SC assembly is complete [37]. We propose that in early zygotene nuclei, Hop1/Red1 and Zip1 are present at Zip3-marked sites (see Figure 3I–3L). During SC polymerization, Zip1 is preferentially deposited in axis regions distal from Zip3, giving rise to the alternating Zip1/Hop1 pattern of pachytene chromosomes. Our results are not compatible with a model of SC assembly where Hop1 is displaced from chromosome axes as Zip1 polymerizes [13]. Hop1 in association with Zip3, is present at substantial levels along pachytene chromosomes, both in WT and in *ndt80Δ*, indicating that Hop1 is an integral component of pachytene chromosomes (this work; [37]).

### Pch2 controls meiotic axis morphogenesis

Pch2 plays key roles in establishing and/or maintaining the distribution of at least two proteins along meiotic chromosome axes. First, Pch2 controls overall levels and localization patterns of Hop1. Accordingly, Hop1-enriched domains appear as multiple discrete foci along WT pachytene chromosomes, while in *pch2Δ*, Hop1 spreads into fewer, more extended structures (Figure S2B, S2C). Importantly, changes in Hop1 loading in *pch2Δ* are not an indirect consequence of e.g. a delay in meiotic progression: In *ndt80Δ* arrested cells, Hop1 forms distinct foci, while in the *ndt80Δpch2Δ* double mutant, Hop1 loads at increased levels and localizes uniformly along chromosomes. Pch2 may control Hop1 association with chromosome axes via its chromosomal localization. Consistent with this idea, in *zip1Δ*, a mutant condition that eliminates Pch2 specifically from chromosome arms, Hop1 and Red1 load in a continuous pattern along chromosome axes, reminiscent of patterns observed in *pch2Δ* [13],[21],[33].

Increased numbers of Zip3 foci along *pch2Δ* pachytene chromosomes further identify a function of Pch2 in controlling association of Zip3 with meiotic chromosome axes. Increased numbers of Zip3 foci in *pch2Δ* may indicate defects in CO designation, possibly indicating an increase in the number of CO designation sites with associated inefficiencies to form functional chiasmata. Notably, Zip3 represents a CO designation marker in WT. In several mutants exhibiting reduced CO levels and loss of CO interference, Zip2 foci form with apparently normal numbers and distribution, indicating that CO designation on the cytological level can be uncoupled from the execution of CO formation [22].

Chromosome axis defects in *pch2Δ* are indicated by increased numbers of Zip3 foci and a failure to undergo appropriate axis shortening. Axis shortening, too, may be an outcome of normal CO designation. Coordinate increases in axis length and number of CO designation sites as well as loss of interference in *pch2Δ* support a linkage between axis length and CO control. SC length and CO numbers are closely correlated in many taxa, including mammals [41],[49], consistent with the idea that CO number and distribution are controlled via the status of the chromosome axis. In a mutant situation such as *pch2Δ*, changes in axis status may indicate defects in chromosome axis status, with possible effects on CO placement and/or formation of functional chiasmata.

### Pch2 controls crossover interference, but is dispensable for CO formation

Pch2 suppresses COs in adjacent chromosome regions without being required for normal CO formation. In *pch2Δ*, map distances tend to be increased when flanking intervals are nonparental, yet are at WT levels when neighboring intervals are parental. The major implication of these results is that functions in CO interference can be separated from those in CO formation. While Pch2 is required for timely CO formation, COs form at normal levels in a *pch2Δ* mutant, both at a hotspot of recombination and in the genetic intervals examined here [35],[37, this work]. Conversely, we consider it as unlikely that Pch2 changes the overall distribution of COs rather than affecting CO interference: An overall change in CO distribution should change individual map distances independent of the presence or absence of a CO in an adjacent interval.

Pch2 further does not exert a general inhibitory effect on recombination: (i) DSBs form at normal levels in *pch2Δ* when analyzed at a recombination hotspot or by a genome-wide approach (A. Hochwagen, personal communication; [35],[37]). (ii) Absence of Pch2 does also not compensate for low DSB levels in hypomorphic *spo11* mutants, e.g. by improving spore segregation. Thus, Pch2 performs a function in CO control without playing a role in overall CO levels.

While CO levels in *pch2Δ* are normal in the intervals examined here, the number of Zip3 foci is substantially increased. We interpret this discrepancy as indicating that CO designation is increased in *pch2Δ*, yet does not result in a corresponding increase in completed COs. At the same time, we cannot exclude that somewhat different incubation conditions result in actual increases in CO levels in *pch2Δ*. Cytological studies presented here were performed in liquid medium, but CO levels along the three chromosomes were determined following sporulation on solid medium. Such minor differences may have major effects on CO levels and distribution in *pch2Δ*. Notably, an independent study from the Alani lab observed increased CO levels in *pch2Δ* (see accompanying paper).

Two classes of COs, one that exhibits interference and the other that does not exhibit interference, have been proposed to contribute to total CO levels in the WT [e.g., 11],[32],[50],[51]. Accordingly, in certain mutants, CO reduction is accompanied by defective interference among residual COs [32]. Identification of *pch2Δ* as a mutant that forms COs at normal levels but is defective for interference suggests that interference is superimposed on basic recombination pathways, and that it can be eliminated without loss of COs.

Absence of Pch2 further results in increased levels of gene conversion. Association of increased gene conversion levels with parental and non-parental configuration of flanking chromosome arms suggests that Pch2 affects this process prior to bifurcation of the CO and NCO pathways. Increased gene conversion without increases in DSB levels could occur due to an increased length of heteroduplexes in ongoing recombination interactions and/or mismatch repair defects in recombination intermediates. Such defects could be an outcome of spatial changes in axis juxtaposition, or due to elimination of other factors.

The biological function of interference is presently mysterious. Several ideas have been put forward to explain this conserved phenomenon [e.g., 52]: (i) Closely spaced double COs provides insufficient sister cohesion resulting in chromosome missegregation [53]. (ii) Interference is a byproduct of the CO assurance system [54]. Linkage between defects in interference, loss of CO assurance and/or normal homolog segregation and reduced spore viability in most yeast mutants with interference defects has complicated our understanding of the function of interference [e.g., 28],[32],[55],[56]. The current study indicates that short range interference is not required for normal chromosome segregation and/or the formation of functional gametes. Notably, all intervals tested along chromosome III exhibit interference defects here, yet chromosome segregation (including chromosome III) is normal in *pch2Δ*, as suggested by high spore viability patterns. Based on this finding, we present a model postulating that interference is a byproduct of the CO assurance system (see below; [54]).

### Role of Pch2 in ensuring spore viability at low DSB levels

Full levels of interference appear dispensable for meiotic chromosome segregation, yet we demonstrate that a mechanism compensating for reduced DSBs is critical for viable gamete formation. Segregation of the 16 homolog pairs is mostly normal during WT meiosis, even when DSBs are reduced to <20% of WT levels (in *spo11da*; this work; [4],[29]). By contrast, in a *pch2Δ* background, DSB reduction to <80% of WT levels (in *spo11yf/spo11-HA*), results in catastrophic reduction of spore viability, identifying essential functions for mechanisms that compensate for reduced DSBs during yeast meiosis.

In a *pch2Δ* mutant hypomorphic for *spo11*, two- and zero viable spore tetrads are highly abundant, a pattern suggestive of defects in homolog disjunction. Such defects are frequently attributed to a failure of homolog pairs to acquire sufficient COs for homolog disjunction. Our analysis of CO levels at the *HIS4LEU2* recombination hotspot does not provide evidence for substantial CO defects in *pch2Δ* at reduced DSB levels. CO levels at *HIS4LEU2* may not be representative for genome-wide CO levels in *pch2Δspo11da*. Notably, unlike other genome regions, *HIS4LEU2* does not exhibit CO homeostasis [29]. Alternatively, COs may form efficiently along the entire genome in *pch2Δspo11da*, but fail to undergo appropriate chiasma maturation. Such defects could affect intersister connections near crossovers resulting in a failure to maintain cohesion along chromosome arms until onset of anaphase. Severe defects in spore viability despite substantial CO formation have also been demonstrated for *pch2Δrad17Δ* double mutants, and may be related to the results reported here [35].

Importantly, results presented here define a Pch2-dependent mechanism that assures homolog segregation at reduced DSB levels. In yeast, on average>five COs/chiasmata form per homolog pair (90 COs distributed among 16 homolog pairs). Accordingly, stabilizing functions in homolog segregation and/or CO assurance may only manifest themselves when initiating DSBs are reduced. In organisms with lower wild-type COs levels, similar defects in chiasma function may result in homolog nondisjunction at normal DSB levels due to a failure to acquire sufficient COs (e.g. the XY pair in mammals [47]).

### Dependence of the *pch2Δ* phenotype on incubation conditions and implications

Unexpectedly, *pch2Δ* defects in CO interference and in spore viability at reduced DSB levels are partially rescued under certain conditions. For example, at 33°C, despite loss of most short range CO interference, some long-range interference (>100 kb) is retained. Furthermore, at 30°C, *pch2Δ* is mostly proficient for interference, yet reduced DSB levels result in formation of inviable spores. Pch2 appears to stabilize CO control and spore viability over a wide range of conditions, including different temperatures and low DSB levels. In the absence of Pch2, a temperature decrease of only 3°C results in catastrophic chromosome missegregation, with no comparable effects in WT highlighting the necessity of Pch2-mediated stabilization of CO control. Oppositely stabilizing and destabilizing effects of temperature on interference and viable spore formation are consistent with the idea that interference and homolog disjunction assuring chiasma formation are the outcome of two antagonistically-acting pathways. Thus, these functions are separable based on their different dependence on incubation temperature. One explanation is that temperature oppositely modulates two chromosome components, e.g. chromosome axes and chromatin fiber (see below).

We infer the existence in the absence of Pch2 of one or several default systems that provide partially functional CO control and/or mechanisms for maintaining high levels of spore viability. Backup systems for CO control may e.g. utilize basic organizational features shared with mitotic chromosomes. Roles in CO control of general structural chromosome components have been demonstrated in *C. elegans* [57].

We note that Pch2-independent backup systems appear to function independent of a properly structured chromosome axis: Incubation conditions modulate *pch2Δ* defects in crossover placement and spore viability/chromosome segregation, but not chromosome axis defects. More uniform Hop1 association in *pch2Δ* occurs over a wide range of conditions, at 33°C and 23°C, and is also evident at 30°C in a different strain background [33]. Drastically different defects in interference and CO homeostasis are observed under the respective conditions (this work; [37]). Together, these data indicate that uniform Hop1 association with chromosome axes is a consequence, not a cause of the initial CO control defect.

### Functions of Pch2 in WT and mutant meiosis

The current work identifies functions of Pch2 during WT meiosis in chromosome morphogenesis, CO placement and spore viability/homolog segregation when DSBs are reduced. In *C. elegans* and *Drosophila*, Pch2 prevents meiotic progression in mutant meiosis when chromosomal events independent of recombination initiation are defective. No role of Pch2 in WT meiosis has been detected in these organisms [34],[38]. In mouse WT meiosis, Pch2 is required for efficient completion of recombination, but no role in mutant meiosis as a checkpoint is apparent [36]. Accordingly, Pch2 has been described as a checkpoint or a factor required for normal meiotic progression.

Pch2 modulates axis status, recombination progression and SC morphogenesis [37, this work]. Thus, Pch2 affects all processes in WT meiosis that it is proposed to monitor as a checkpoint during mutant conditions. We propose that control of chromosome axis status constitutes Pch2's primary function, with secondary effects on recombination progression, CO placement and homolog segregation. Accordingly, changes in axis status may result in destabilized homolog juxtaposition, with downstream effects such as delayed double Holliday junction turnover, delayed CO/NCO formation and aberrantly high levels of non-Mendelian segregation events [37, this work]. *pch2Δ* induced changes in axis status would likely also affect axis-associated processes under mutant conditions, with possible consequences for checkpoint activation. Modulation of underlying defects rather than compromised monitoring represents an attractive explanation for diverse Pch2 functions in mutant and WT meiosis. Alternatively, Pch2 may perform unrelated functions in meiotic cell cycle control, CO placement and gamete viability.

We note that in yeast, *pch2Δ* defects in WT meiosis are relatively subtle, and detectable only under certain conditions (see above). Corresponding defects in other organisms may also be difficult to detect and/or become manifest only under certain conditions. Consistent with this idea, in *Drosophila*, a synergistic effect of *pch2Δ* on CO levels has recently been demonstrated in combination with another mutant [38].

### Model: Pch2 as a one-crossover domain size determinant

One key outcome of the current study is that Pch2 appears to suppress or enhance formation of functional chiasmata at normal or reduced DSB levels, respectively. Here, we propose a model integrating these apparently opposing functions of Pch2. Pch2 is proposed to reorganize chromosome axes into long range CO control modules, hereafter referred to as ‘One Crossover Modules’ (OCMs). Key features of OCMs include assurance to undergo one CO, and suppression of additional COs within the same module. Modules are proposed to tile each bivalent, resulting in formation of as many COs as OCMs.

Pch2-mediated CO control is proposed to occur in two-steps: (i) bivalents become organized into a tiling array of OCMs. (ii) CO designation and interference occur. Cytologically, each OCM would correspond to a centrally localized Zip3 focus with associated Hop1 hyperabundance domain extending to both sides into Hop1-poor regions, reflecting the reach of interference. Pch2 is proposed to function as a determinant for OCM installation.

The stress hypothesis of CO control provides a mechanistic explanation of how chiasma assurance/maturation and interference might be linked along each OCM [27],[54]. We propose that each OCM constitutes an independent stress module. Stress and stress relief along the axis are hypothesized to mediate crossover designation and interference, respectively. Specifically, compression stress along the axis would result in localized axis deformation with two important consequences, stress relief and CO designation. Stress relief prevents additional axis deformation events along each OCM, effectively establishing interference.

By setting a module for stress transmission, Pch2 would promote CO progression of a DSB proximal to the deformed axis segment, and coordinately prevent additional DSBs from undergoing the same fate. OCMs may become installed *de novo*, or, more likely, be specified via modification of preexisting chromosome features. Available data are easily integrated with this model: Pch2 associates with chromosome axes during the early zygotene stage. At the same stage, CO/NCO differentiation is finalized, axis domains associated with future COs appear, and the interference distribution of Zip2/Zip3 becomes established [9],[11],[22]. Programmed axis deformation at CO sites associated with stress relief and CO designation could further contribute to axis shortening. When defective, this may result in aberrantly long axes (this work). Moreover, axis deformation may promote assembly of Red1/Hop1 and Zip3 at CO-designated sites, with aberrant CO designation/axis deformation resulting in uniform Hop1 axis association.

Default modules that provide some CO control in *pch2Δ* may result in suboptimal CO designation, aberrant CO positioning, or increased sensitivity to incubation conditions. Such effects may be particularly detrimental when DSBs are limiting. Under such conditions, DSBs normally ensured to become COs may now fail to induce steps in chromosome morphogenesis associated with normal chiasma formation.

Control of CO numbers via one-crossover modules provides an attractive way how recombination frequencies can be controlled in different organisms and even different sexes. Accordingly, in *C. elegans*, each chromosome would be organized as a single module, while in e.g. mouse, there would be one or two OCMs per homolog pair. In many species, CO levels between males and females differ for identical homolog pairs. Setting differently sized OCMs represent an attractive way to control CO levels in a chromosome-wide manner. Levels and distribution of COs are dramatically modulated by temperature and other environmental factors in many eukaryotes (e.g., [58]). Such sensitivity along WT chromosomes may be related to the mutant sensitivities revealed by the current work.

### Concluding remarks

In summary, we have demonstrated here that chromosome axes undergo programmed changes in their global structure that strikingly parallel the non-random positioning of chiasmata during meiosis. Unlike other cases of cytologically detectable chromosome domain organization, including heterochromatin assembly, such domainal organization is determined individually for each cell, in accordance with non-random meiotic crossover distribution. Close functional and temporal coordination between assured crossover formation and chromosome domain organization identify potential functions for chromosome axis status in faithful meiotic homolog segregation.

## Materials and Methods

### Yeast strains

Strains were of the SK1 background (Table S5). Markers were introduced by transformation or crossing and were verified by Southern blot. N-terminally HA-tagged *PCH2* was transferred from the BR strain background [33] by insertion of the *URA3* marker 300 bp upstream of *PCH2* followed by PCR amplification of the tagged construct including the marker and transformation into SK1 (strain gift from A. Hochwagen). In the resulting strain, the 3×HA-tag encoding sequence is flanked by 15 and 14 polylinker-encoded amino acids, respectively (N.J., unpublished data).

### Tetrad analysis of recombination on chromosomes III, VII, and VIII

Haploids mated overnight on supplemented YPD were transferred to identical batches of sporulation medium (0.5% potassium acetate, 0.02% raffinose) and incubated at 33°C or 30°C for 72 hrs. Asci were incubated with zymolyase, dissected on supplemented YPD and replica-printed to appropriate media to determine marker status. Tetrads exhibiting non-Mendelian segregation of ≥5 markers were assumed to be false tetrads and omitted from further analysis. For calculations of map distances and NPD frequencies, see text. Standard error calculations were performed using the Stahl Lab Online Tools. Tetrads with non-Mendelian segregation for either marker of an interval were omitted for calculations for that interval. Chi square values were used to calculate P-values using the Vassar College webpage.

### Immunocytology, focus scoring, and statistical analysis

Time courses and meiotic spreads were prepared and immunostained as described [11]. Chromatin was stained using DAPI. Hop1 and Zip1 were stained with rabbit anti-Hop1 (F. Klein) and rabbit anti-Zip1 (S. Keeney) antibodies at 1∶300 to 1∶1000, except for nuclei shown in Figure 4Q–4V in which mouse anti-Zip1 antibody (P. Moens) was used at 1∶500 dilution. GFP- and HA fusion proteins were detected with goat anti-GFP (Rockland) at 1∶400 or mouse anti-HA (Covance) antibodies at 1∶1000 dilution, followed by incubation secondary antibodies conjugated to Alexa 488-, Alexa 594-, or Alexa 680 (Molecular Probes) at 1∶2500 dilution. All antibodies were tested for epitope specificity using appropriate deletion/untagged strains. Images were captured by a computer-assisted fluorescence microscope system (DeltaVision, Applied Precision). The objective lens was an oil-immersion lens (100×, NA = 1.35). Image deconvolution was carried out using an image workstation (SoftWorks; Applied Precision). In double staining experiments, real colocalization was scored by counting the number of overlapping foci in composite images. Fortuitous colocalization was evaluated by the misorientation method where one of the two images is rotated by 180°, ensuring maximum nucleus overlap, and colocalization is determined by counting the number of overlapping foci [59].

ImageJ was used for processing and quantitative analysis of images saved as 16bit TIFF files in SoftWorks. To analyze Hop1 distribution patterns, threshold levels were visually adjusted to maximize signal detection within the DAPI staining area, followed by measurements of the Hop1 positive area and the mean signal intensities at above background levels. Subsequently, threshold levels were set to the mean signal intensity for each image, and a mask was generated for the Hop1 positive signals exhibiting≥average signal intensities. Masks were transferred into MicroMeasure to determine the number and maximum lengths of individual Hop1 signals (below). Total SC length in WT and *pch2Δ* strains were measured by a “blind” observer, using MicroMeasure. MStat 5.1 was used for data plotting and statistical analysis.

### Web resources

Rasband, WS, ImageJ, U. S. National Institutes of Health, Bethesda, Maryland, USA, http://rsb.info.nih.gov/ij/, 1997–2008

Drinkwater N Mstat Statistical Software: http://mcardle.oncology.wisc.edu/mstat/

Stahl Lab Online Tools: http://www.molbio.uoregon.edu/~fstahl/

MicroMeasure, version 3.3: (http://www.colostate.edu/Depts/Biology/MicroMeasure

## Supporting Information

Figure S1 Meiotic events in a synchronized time course expressing HA-Pch2. Stages of cells were determined in spread nuclei based on staining with an antibody against Zip1. Zip1-staining was classified as unstained nuclei (presumably corresponding to pre-leptotene or post-pachytene cells), leptonema (foci), zygonema (foci plus short lines) or pachynema (mostly lines with little or no foci). Nuclear divisions are determined in DAPI-stained samples from the same time course, ≥2 indicates completion of meiosis I and/or II.
(0.37 MB EPS)

Figure S2 Hop1 domains in *PCH2ndt80Δ* and *pch2Δndt80Δ*. (A) Mean pixel intensities of Hop1 signals were determined by image quantitation in ImageJ, following threshold adjustment for maximum signal detection. Intensities are given in arbitrary units (AU). p<0.0001 (B) Number of Hop1 domains exhibiting at or above average intensity. Note that this analysis omits weaker Hop1 positive chromosome regions included in the WT analysis. (C) Length measurements of individual Hop1 signals detected in 20 *PCH2ndt80Δ* (grey) and 20 *pch2Δndt80Δ* (red) nuclei, respectively, ordered by size. Each dot corresponds to a structure measured: 745 Hop1 signals were measured in *PCH2ndt80Δ* and 531 Hop1 signals in *pch2Δndt80Δ*. Average length of Hop1 structures is 0.73 µm (±0.02 µm S.E.) and 1.44 µm (±0.05 µm S.E.) in *PCH2ndt80Δ* and *pch2Δndt80Δ*, respectively (p<0.0001). All signal intensities in the examined nuclei are within the linear range of detection. p-values were determined using the two-sided Wilcoxon rank sum test in MStat. Error bars represent 95% confidence intervals (±1.96*SE).
(0.73 MB EPS)

Figure S3 Number of COs per chromosome in WT and *pch2Δ* at 33°C. No increase in tetrads exhibiting zero COs is observed. Goodness of fit tests for *pch2Δ* versus WT give P-values of <0.0001 (chromosome III), 0.0016 (chromosome VII), and 0.2328 (chromosome VIII). Significant deviations from the WT along chromosomes III and VII are likely due to increase of tetrads with three or more COs per chromosome in *pch2Δ*. Numbers of COs per tetrad for a given chromosome were determined by sorting printed versions of the tetrads according to the number of COs. Significance for the tetrad classes exhibiting zero, one, two, and three COs was determined using the Vassar statistics website (see Materials and Methods).
(0.71 MB TIF)

Figure S4 Crossovers and noncrossovers on chromosome arms carrying non-Mendelian segregation/gene conversion events in WT and *pch2Δ* at 33°C. Internal markers exhibiting gene conversions were selected and flanking intervals were categorized as parental, recombinant, or non-Mendelian.
(0.58 MB TIF)

Figure S5 Genetic map distances in WT and *pch2Δ* at 30°C. Genetic distances determined for intervals 1–9 (see Figure 5A). Contributions of tetratypes (TT) and nonparental ditypes (NPD) to map distances are indicated in different shades. Error bars represent standard errors (see Table S4 for numbers of valid tetrads for each interval). No significant differences between map distances in WT and *pch2Δ* strains were detected.
(0.58 MB TIF)

Table S1 Crossover interference on three chromosomes in wild-type and *pch2Δ* at 33°C and 30°C.
(1.09 MB PDF)

Table S2 Non-Mendelian segregation in WT and *pch2Δ* tetrads at 33°C and 30°C.
(1.05 MB PDF)

Table S3 Spore viabilities in WT and *pch2Δ* strains carrying *spo11* hypomorphic mutations.
(1.05 MB PDF)

Table S4 Effects of *pch2Δ* at 30°C on genetic distances and crossover interference in intervals along three chromosomes.
(0.06 MB DOC)

Table S5 *S. cerevisiae* strains used in this study.
(0.04 MB DOC)
