# Supplementary figures and images for: Pch2 Links Chromosome Axis Remodeling at Future Crossover Sites and Crossover Distribution during Yeast Meiosis
Source: PLoS Genet. 2009 Jul 24;5(7):e1000557. doi: 10.1371/journal.pgen.1000557 (PMC2708914; doi:10.1371/journal.pgen.1000557)

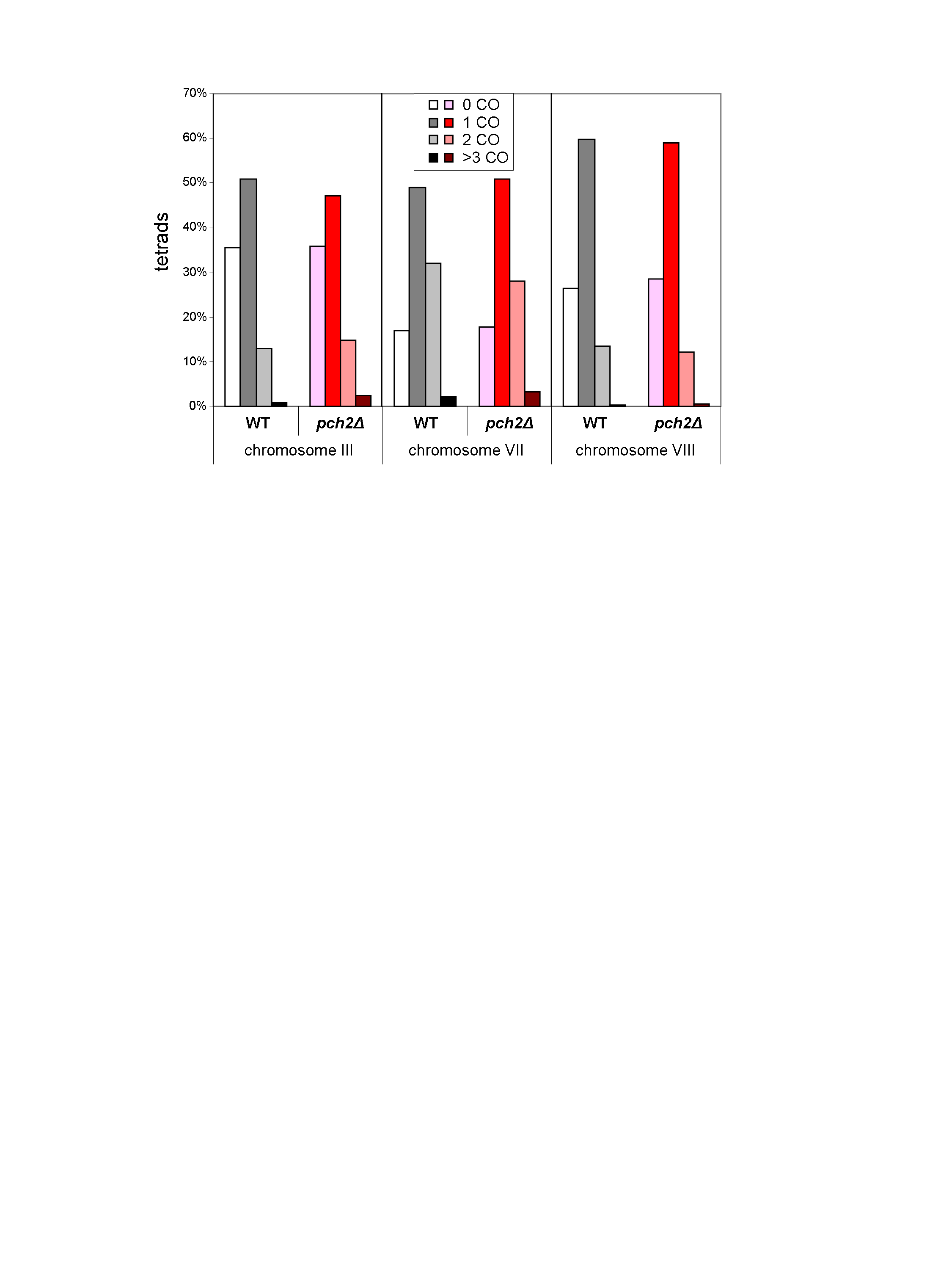

Supplement: Figure S3 — Number of COs per chromosome in WT and pch2Δ at 33°C. No increase in tetrads exhibiting zero COs is observed. Goodness of fit tests for pch2Δ versus WT give P-values of <0.0001 (chromosome III), 0.0016 (chromosome VII), and 0.2328 (chromosome VIII). Significant deviations from the WT along chromosomes III and VII are likely due to increase of tetrads with three or more COs per chromosome in pch2Δ. Numbers of COs per tetrad for a given chromosome were determined by sorting printed versions of the tetrads according to the number of COs. Significance for the tetrad classes exhibiting zero, one, two, and three COs was determined using the Vassar statistics website (see Materials and Methods). (0.71 MB TIF) [file pgen.1000557.s003.tif]

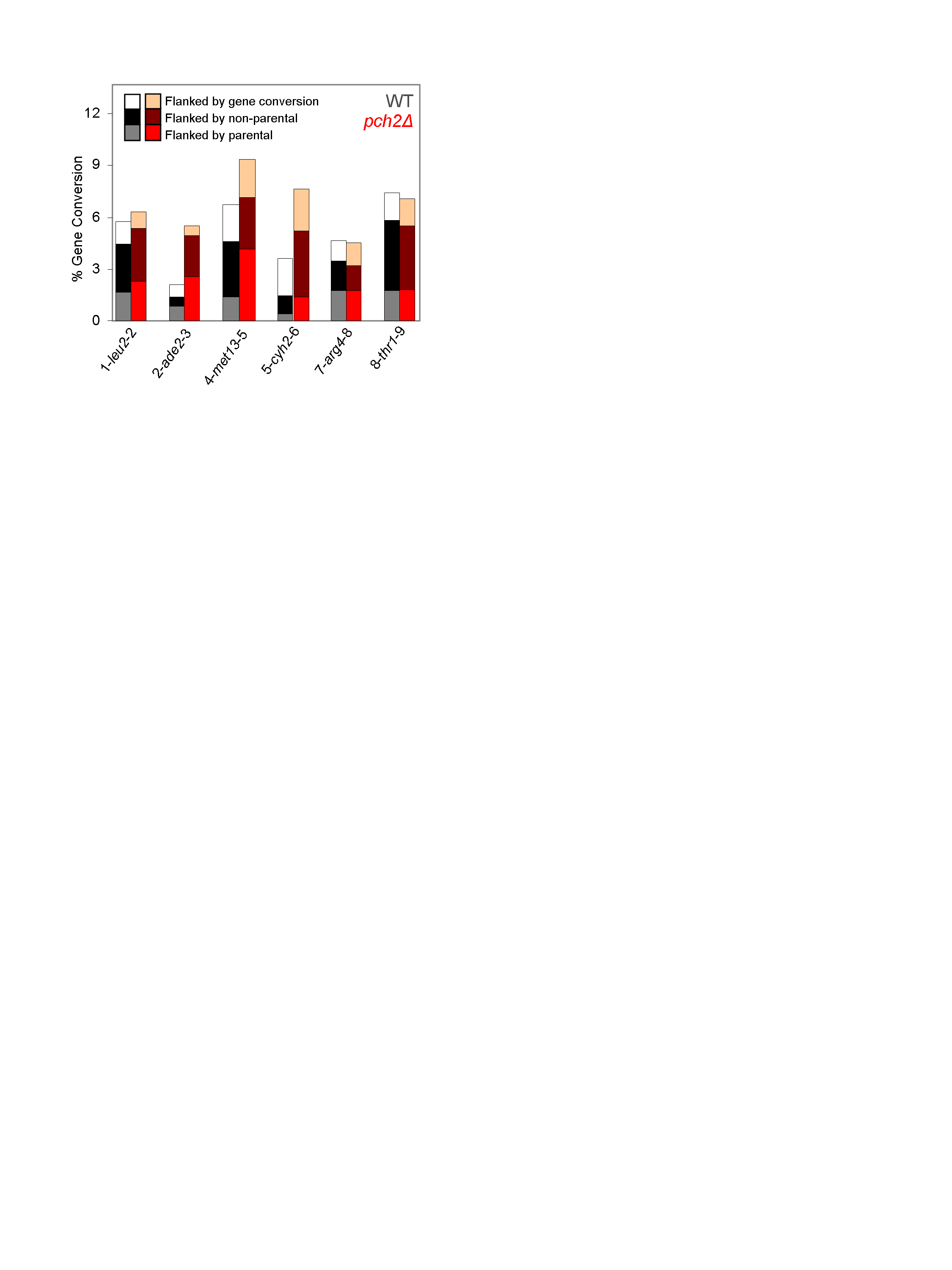

Supplement: Figure S4 — Crossovers and noncrossovers on chromosome arms carrying non-Mendelian segregation/gene conversion events in WT and pch2Δ at 33°C. Internal markers exhibiting gene conversions were selected and flanking intervals were categorized as parental, recombinant, or non-Mendelian. (0.58 MB TIF) [file pgen.1000557.s004.tif]

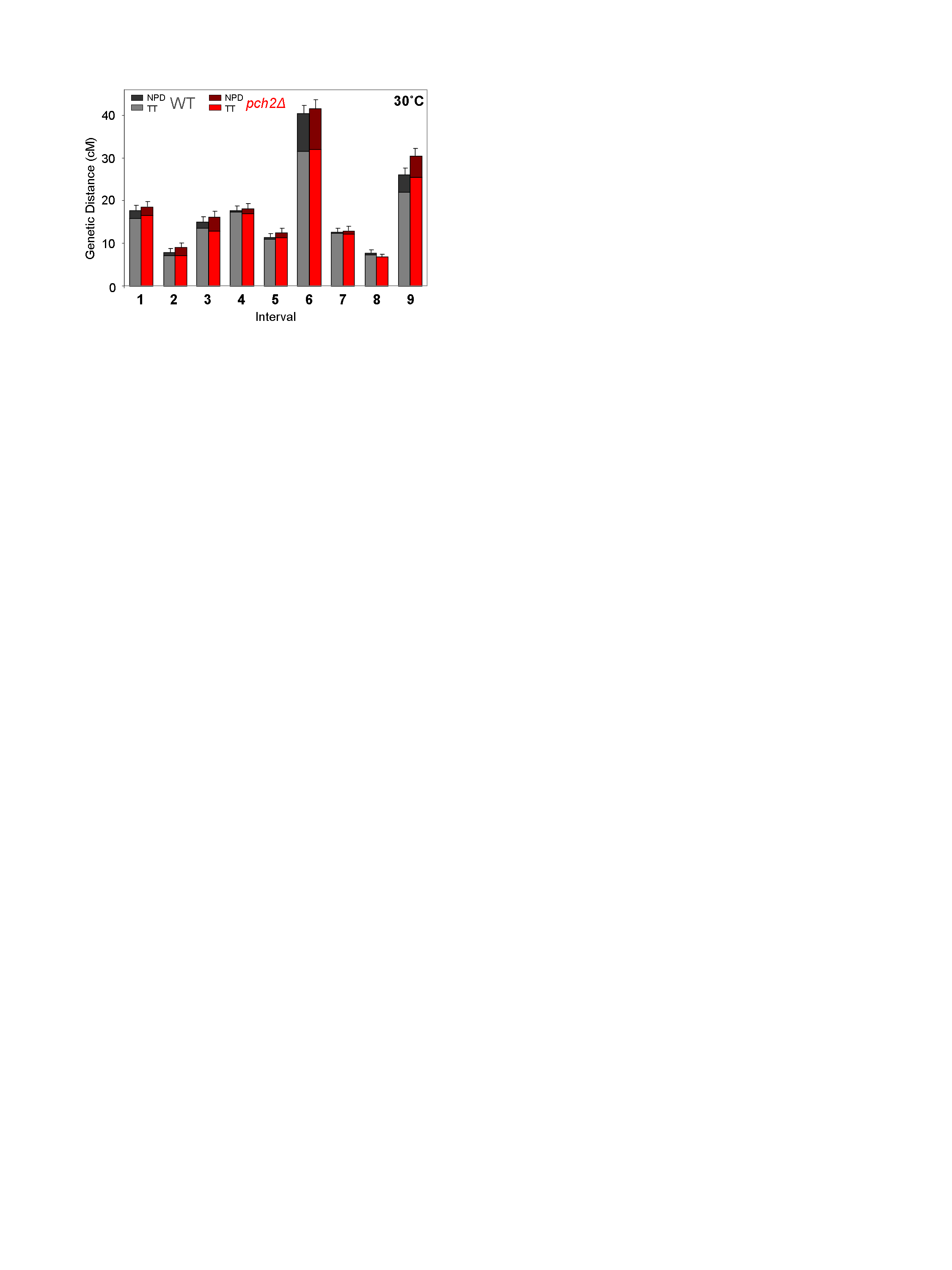

Supplement: Figure S5 — Genetic map distances in WT and pch2Δ at 30°C. Genetic distances determined for intervals 1–9 (see Figure 5A). Contributions of tetratypes (TT) and nonparental ditypes (NPD) to map distances are indicated in different shades. Error bars represent standard errors (see Table S4 for numbers of valid tetrads for each interval). No significant differences between map distances in WT and pch2Δ strains were detected. (0.58 MB TIF) [file pgen.1000557.s005.tif]
