# Supplementary material for: Pch2 Links Chromosome Axis Remodeling at Future Crossover Sites and Crossover Distribution during Yeast Meiosis
Source: PLoS Genet. 2009 Jul 24;5(7):e1000557. doi: 10.1371/journal.pgen.1000557 (PMC2708914; doi:10.1371/journal.pgen.1000557)
Supplement: Table S1 — Crossover interference on three chromosomes in wild-type and pch2Δ at 33°C and 30°C. (1.09 MB PDF) [file pgen.1000557.s006.pdf]

Table S1. Crossover interference on three chromosomes in wild-type and *pch2Δ* at 33°C and 30°C.

A. Chromosome III

Genotype; Temperature	Reference Interval / Test Interval		*his4-leu2*		*leu2-CEN3*		*CEN3-MAT*	
			leu2-CEN3	*CEN3-MAT*	*his4-leu2*	*CEN3-MAT*	*his4-leu2*	*leu2-CEN3*
Wild type; 33°C	P	P:N:T	528:0:183	469:3:222	528:4:297	578:4:264	467:4:219	578:0:142
		cM ± SE	12.9±0.8	17.3±1.1	19.4±1.1	17.0±.9	17.6±1.2	9.9±.7
	T+N	P:N:T	301:0:19	226:1:87	183:1:18	142:0:54	225:0:87	268:0:5
		cM ± SE	3.0±0.7	14.8±1.6	5.9±1.8	13.8±1.6	13.9±1.31	.92±.41
	ratio		.23±.05	.86±.11	.31±.09	.81±.11	.79±.09	.09±.04
	sig (SE) †		S	N	S	N	S	N
	sig (p) *		9.35E-3	.380	.018	.093	.037	0.116
pch2Δ/”; 33°C	P	P:N:T	509:3:154	402:10:238	509:10:283	547:6:298	403:8:220	547:1:146
		cM ± SE	12.9±1.1	22.9±1.7	21.3±1.4	19.6±1.2	21.2±1.6	11.0±0.9
	T+N	P:N:T	297:1:33	234:3:97	159:5:31	148:8:61	250:7:89	304:3:64
		cM ± SE	5.9±1.2	17.2±1.9	15.6±3.6	25.1±4.0	18.9±2.5	11.0±1.7
	ratio		.46±.10	.75±.10	.73±.17	1.28±.21	.89±.02	1.01±.17
	sig (SE) †		S	S	N	N	N	N
	sig (p) *		2.16E-3	.041	.128	.183	.145	.124
	sig (ratios)‡		N	N	S	S	N	S
Wild type; 30°C	P	P:N:T	426:0:100	369:1:145	426:4:232	482:3:195	366:3:179	482:1:91
		cM ± SE	9.5±0.9	14.6±1.1	19.3±1.3	15.7±1.1	18.0±1.3	8.5±0.9
	T+N	P:N:T	238:2:13	185:2:63	100:1:14	92:1:25	146:2:63	198:1:25
		cM ± SE	4.9±1.8	15.0±2.1	8.7±3.0	13.1±3.1	17.8±2.5	6.9±1.7
	ratio		.52±.19	1.02±.16	.45±.16	.84±.21	.99±.16	.82±.22
	sig (SE) †		S	N	S	N	N	N
	sig (p) *		3.80E-2	.630	6.86E-5	.100	.988	.189
pch2Δ/”; 30°C	P	P:N:T	396:2:87	338:5:136	396:3:221	475:6:167	337:2:186	475:4:80
		cM ± SE	10.2±1.2	17.3±1.67	19.3±1.2	15.7±1.3	18.8±1.3	9.3±1.3
	T+N	P:N:T	228:2:17	195:3:49	90:1:19	85:2:28	142:1:51	173:1:29
		cM ± SE	5.9±1.9	13.6±2.4	11.3±3.2	17.4±4.0	14.6±2.2	8.6±2.0
	ratio		.58±.20	.78±.16	.59±.16	1.1±.28	.78±.12	.93±.24
	sig (SE) †		N	N	S	N	N	N
	sig (p) *		.406	.112	.019	.171	.230	.39
	sig (ratios)‡		N	N	N	N	N	N

B. Chromosome VII

Genotype; Temperature	Reference Interval / Test Interval		*lys5-met13*		*met13-cyh2*		*cyh2-trp5*	
			met13-cyh2	*cyh2-trp5*	*lys5-met13*	*cyh2-trp5*	*lys5-met13*	*met13-cyh2*
Wild type; 33°C	P	P:N:T	485:0:169	211:14:409	485:5:352	297:18:521	211:2:153	297:0:80
		cM ± SE	12.9±0.9	38.9±1.8	22.7±1.0	37.6±1.6	22.5±1.7	10.6±1.0
	T+N	P:N:T	357:1:28	155:6:221	169:0:29	80:2:119	422:2:224	539:1:119
		cM ± SE	4.4±1.0	33.6±2.1	7.3±1.3	32.6±2.6	18.2±1.1	9.5±0.9
	ratio		.34±.08	.87±.07	.32±.06	.87±.08	.81±.08	.89±.12
	sig (SE) †		S	N	S	N	S	N
	sig (p) *		4.42E-3	.753	5.79E-14	.103	1.00E-6	.17
pch2Δ/" 33°C	P	P:N:T	466:1:142	230:16:335	466:2:322	283:25:475	230:2:148	283:1:112
		cM ± SE	12.2±1.0	37.0±2.1	21.1±1.0	39.9±1.9	21.0±1.6	14.9±1.3
	T+N	P:N:T	324:1:53	153:12:202	143:2:50	115:5:84	433:1:263	606:0:116
		cM ± SE	7.8±1.2	37.3±2.8	15.9±2.6	27.9±3.5	19.3±0.0	8.0±0.7
	ratio		.64±.11	1.00±.09	.75±.13	.70±.09	.92±.09	.54±.07
	sig (SE) †		S	N	N	S	N	S
	sig (p) *		1.49E-2	.870	.057	.051	.164	7.02E-2
	sig (ratios)‡		S	N	S	N	N	S
Wild type; 30°C	P	P:N:T	353:0:144	150:21:313	353:0:242	185:18:391	151:0:110	185:0:83
		cM ± SE	14.5±1.0	45.4±2.7	20.3±1.0	42.0±2.1	21.1±1.5	15.5±1.4
	T+N	P:N:T	243:1:25	111:4:153	144:1:25	83:8:79	333:1:154	409:1:85
		cM ± SE	5.8±1.4	33.0±2.5	9.1±2.2	37.4±4.9	16.4±1.2	9.2±1.0
	ratio		.40±.10	.73±.07	.45±.11	.89±.12	.78±.08	.59±.09
	sig (SE) †		S	S	S	N	S	S
	sig (p) *		1.14E-3	.007	1.86E-9	.143	5.22E-4	.015
pch2Δ/"; 30°C	P	P:N:T	341:1:124	155:22:278	341:2:203	188:21:343	153:1:84	188:1:60
		cM ± SE	14.0±1.2	45.5±2.9	19.7±1.3	42.5±2.4	18.9±2.0	13.3±1.8
	T+N	P:N:T	206:2:32	87:3:153	125:1:33	64:5:96	298:2:149	364:2:99
		cM ± SE	9.2±2.0	35.2±2.4	12.3±2.4	38.2±4.1	17.9±1.4	11.9±1.3
	ratio		.66±.16	.78±.07	.62±.13	.90±.11	.95±.12	.90±.16
	sig (SE) †		S	S	S	N	N	N
	sig (p) *		1.37E-29	.049	.035	.419	.126	.050
	sig (ratios)‡		N	N	N	N	N	S

C. Chromosome VIII

Genotype; Temperature	Reference Interval / Test Interval		*CEN8-arg4*		*arg4-thr1*		*thr1-cup1*	
			arg4-thr1	*thr1-cup1*	*CEN8-arg4*	*thr1-cup1*	*CEN8-arg4*	*arg4-thr1*
Wild type; 33°C	P	P:N:T	629:1:159	347:8:395	626:2:232	371:7:446	349:2:136	371:0:118
		cM ± SE	10.5±0.8	29.5±1.4	14.2±0.9	29.6±1.2	15.2±1.3	12.1±1.0
	T+N	P:N:T	234:0:6	138:0:89	163:1:5	118:1:43	407:1:92	459:1:43
		cM ± SE	1.2±0.5	19.6±1.6	3.2±1.9	15.1±2.5	9.8±1.0	4.9±0.9
	ratio		.12±.05	.66±.06	.23±.13	.51±.09	.64±.09	.40±.08
	sig (SE) †		S	S	S	S	S	S
	sig (p) *		9.75E-3	1.98E-8	1.93E-5	.002	4.72E-3	1.13E-3
pch2Δ/”; 33°C	P	P:N:T	681:1:167	332:10:412	681:2:220	363:9:452	363:0:112	332:2:133
		cM ± SE	10.2±0.8	31.3±1.5	12.9±0.8	30.7±1.3	11.8±0.9	15.5±1.4
	T+N	P:N:T	224:3:10	137:0:80	168:3:10	112:1:48	468:3:47	427:2:83
		cM ± SE	5.9±2.3	18.4±1.6	7.7±3.0	16.8±2.5	6.3±1.2	9.3±1.1
	ratio		.58±.23	.59±.06	.60±.23	.55±.09	.53±.11	.60±.09
	sig (SE) †		N	S	N	S	S	S
	sig (p) *		.237	.004	.473	.049	2.68E-3	6.35E-11
	sig (ratios)‡		S	N	N	N	N	N
Wild type; 30°C	P	P:N:T	473:0:100	265:11:274	473:0:180	306:11:310	306:1:93	264:1:136
		cM ± SE	8.7±0.8	30.9±1.9	13.8±0.8	30.0±1.7	12.4±1.3	17.7±0.9
	T+N	P:N:T	180:1:13	137:0:48	101:1:13	95:0:14	323:0:14	290:0:50
		cM ± SE	4.9±1.8	13.0±1.6	8.3±2.9	6.4±1.6	2.1±2.4	7.4±1.6
	ratio		.56±.21	.42±.06	.60±.22	.21±.05	.17±.05	.42±.06
	sig (SE) †		N	S	N	S	S	S
	sig (p) *		.634	4.98E-13	.166	2.00E-15	.050	8.03E-9
pch2Δ/”; 30°C	P	P:N:T	486:0:85	219:12:228	486:2:173	273:13:321	219:0:112	273:0:65
		cM ± SE	7.4±0.7	32.7±2.3	14.0±1.1	32.9±1.9	16.9±1.3	9.6±1.1
	T+N	P:N:T	175:0:18	112:1:64	85:0:18	65:0:32	308:2:66	341:0:33
		cM ± SE	4.6±1.1	19.8±2.4	8.7±1.9	16.5±2.4	10.4±1.5	4.4±0.7
	ratio		.62±.15	.60±.09	.62±.14	.50±.08	.61±.10	.46±.09
	sig (SE) †		S	S	S	S	S	S
	sig (p) *		.012	2.65E-13	.014	3.09E-19	2.44E-12	2.38E-8
	sig (ratios)‡		N	S	N	S	S	N

Table S1, continued. Crossover interference for tetrads was analyzed by coincidence analysis as described by Malkova et al. (2004). For each test interval, tetrads were divided into two groups based on the crossover status of the reference interval, carrying a parental ditype (P) or a tetratype or nonparental ditpye (T + N), respectively. For each subset of tetrads, map distances and standard errors of test intervals were determined in centiMorgan (cM ± standard error) as described in Materials and Methods.
The "ratio" of the two map distances is taken as an indication of the strength of interference between the reference and the test interval: The lower the ratio, the stronger the interference. Standard errors for ratios are calculated using the application "Analysis of Statistical Significance of Differences Between Two Ratios of Map Distances" from the Stahl website (see Materials and Methods).
Statistical significances are determined in two ways. First, if the absolute value of the difference between the two map distances is greater than twice the standard error, the difference was considered as significant. Second, the distribution of PD, NPD, and TT tetrads was assessed by *G* test. Differences with $p<0.05$ indicate deviation from the expected distribution of these three tetrad classes, indicating crossover interference.

† Significance of difference in ratios between reference and test intervals as calculated by comparing standard errors.
* Significance of difference in ratios between reference and test intervals as calculated by G test using the site at http://faculty.vassar.edu/lowry/VassarStats.html with information from the site http://udel.edu/~mcdonald/statgtestgof.html.
‡ Significances of the differences between ratios between wild-type and *pch2Δ* as calculated by comparing standard errors.
