# Supplementary material for: Pch2 Links Chromosome Axis Remodeling at Future Crossover Sites and Crossover Distribution during Yeast Meiosis
Source: PLoS Genet. 2009 Jul 24;5(7):e1000557. doi: 10.1371/journal.pgen.1000557 (PMC2708914; doi:10.1371/journal.pgen.1000557)
Supplement: Table S2 — Non-Mendelian segregation in WT and pch2Δ tetrads at 33°C and 30°C. (1.05 MB PDF) [file pgen.1000557.s007.pdf]

Table S2. Non-Mendelian Segregation in WT and *pch2Δ* tetrads at 33˚C and 30˚C.

Genotype; Temperature		*his4*	*leu2*	*CEN3:: ADE2*	*MAT*	*lys5*	*met13*	*cyh2*	*trp5*	*CEN8:: URA3*	*arg4*	*thr1*	*cup1*
Wild type;	3:1,1:3	28,39	42,23	8,15	15,26	19,28	38,32	12,26	28,12	8,8	35,18	36,46	21,31
33°C	Other*	2	2	1	2	2	8	3	1		1	4	10
Freq.		.058	.056	.020	.035	.040	.060	.033	.034	.014	.046	.071	.045
pch2Δ/”;	3:1,1:3	26,106	61,18	8,56	7,31	14,82	89,26	18,79	63,22	22,6	34,22	39,46	23,110
33°C	Other*	5	4	4	1	5	3	1	3	2	2	3	8
Freq.		.107	.064	.052	.031	.077	.093	.078	.069	.023	.045	.069	.107
Wild type;	3:1,1:3	20,27	13,20	7,3	13,10	14,20	20,33	12,22	14,15	0,7	8,24	29,33	19,26
30°C	Other*	3				5	2	2	1		1	2	4
Freq.		.055	.039	.012	.027	.040	.062	.040	.034	.008	.037	.073	.053
pch2Δ/”;	3:1,1:3	16,39	29,12	1,27	9,5	14,44	48,18	13,34	32,10	7,6	15,12	28,32	14,58
30°C	Other*	9	1	4		2	6		4	1			13
Freq.		.065	.048	.033	.017	.068	.078	.055	.050	.015	.032	.071	.085

Number of tetrads with 3:1 or 1:3 segregation are shown, along with the frequency of non-Mendelian segregation for the specified locus.

Other* 4,0 or 0,4 segregation.
