# Supplementary material for: Pch2 Links Chromosome Axis Remodeling at Future Crossover Sites and Crossover Distribution during Yeast Meiosis
Source: PLoS Genet. 2009 Jul 24;5(7):e1000557. doi: 10.1371/journal.pgen.1000557 (PMC2708914; doi:10.1371/journal.pgen.1000557)
Supplement: Table S3 — Spore viabilities in WT and pch2Δ strains carrying spo11 hypomorphic mutations. (1.05 MB PDF) [file pgen.1000557.s008.pdf]

Table S3. Spore viabilities in WT and *pch2Δ* strains carrying *spo11* hypomorphic mutations.

SPO11 Genotype	*PCH2* Genotype	Temp. (°C)	4:0	3:1	2:2	1:3	0:4	Total	Viability (%)
Wild-type	*Wild-type*	30	880	43	10	2	0	935	98.2
HA/HA	*Wild-type*	30	77	14	7	2	0	100	91.5
yf-HA/HA	*Wild-type*	30	80	9	2	8	1	100	89.8
da-HA/da-HA	*Wild-type*	30	58	3	10	0	29	100	65.3
Wild-type	*Δpch2*	30	862	64	15	0	0	941	97.5
HA/HA	*Δpch2*	30	74	13	6	7	0	100	88.5
yf-HA/HA	*Δpch2*	30	18	2	6	2	72	100	23.0
da-HA/da-HA	*Δpch2*	30	8	11	19	5	57	100	27.0
Wild-type	*Wild-type*	33	1207	208	41	5	0	1461	82.6
HA/HA	*Wild-type*	33	85	4	10	1	0	100	93.0
yf-HA/HA	*Wild-type*	33	64	12	11	0	10	97	80.0
da-HA/da-HA	*Wild-type*	33	50	11	14	7	18	100	67.0
Wild-type	*Δpch2*	33	1279	203	39	2	0	1523	84.0
HA/HA	*Δpch2*	33	84	3	12	1	0	100	92.5
yf-HA/HA	*Δpch2*	33	75	4	18	2	1	100	87.5
da-HA/da-HA	*Δpch2*	33	33	20	18	14	5	90	67.0

Spore viability pattern, live:dead (no. tetrads)
