# Supplementary material for: Pch2 Links Chromosome Axis Remodeling at Future Crossover Sites and Crossover Distribution during Yeast Meiosis
Source: PLoS Genet. 2009 Jul 24;5(7):e1000557. doi: 10.1371/journal.pgen.1000557 (PMC2708914; doi:10.1371/journal.pgen.1000557)
Supplement: Table S4 — Effects of pch2Δ at 30°C on genetic distances and crossover interference in intervals along three chromosomes. (0.06 MB DOC) [file pgen.1000557.s009.doc]

**Table S4.**  Effects of *pch2Δ* at 30˚C on genetic distances and crossover interference in intervals along three chromosomes.

|  |  | Chromosome III | | | Chromosome VII | | | Chromosome VIII | | |
| --- | --- | --- | --- | --- | --- | --- | --- | --- | --- | --- |
|  |  |  |  |  |  |  |  |  |  |  |
| Genotype |  | *his4-leu2* | *leu2-CEN3* | *CEN3-MAT* | *lys5-met13* | *met13-cyh2* | *cyh2-trp5* | *CEN8-arg4* | *arg4-thr1* | *thr1-cup1* |
| Interval name |  | (1) | (2) | (3) | (4) | (5) | (6) | (7) | (8) | (9) |
|  |  |  |  |  |  |  |  |  |  |  |
| Wild type | P:N:T | 529:5:247 | 698:2:118 | 596:4:225 | 506:1:270 | 610:1:172 | 282:26:489 | 616:1:202 | 656:1:113 | 415:11:329 |
|  |  |  |  |  |  |  |  |  |  |  |
|  | cM | 17.7±1.2 | 7.9±0.8 | 15.1±0.4 | 17.8±0.9 | 11.4±0.8 | 40.5±1.9 | 12.7±0.8 | 7.7±0.7 | 26.2±1.5 |
|  |  |  |  |  |  |  |  |  |  |  |
|  | P* | .041 | .820 | .086 | <.0001 | .056 | <.0001 | .019 | .399 | .005 |
|  |  |  |  |  |  |  |  |  |  |  |
| *pch2Δ*/” | P:N:T | 496:5:247 | 659:5:111 | 588:9:205 | 481:3:248 | 570:3:167 | 263:27:471 | 609:2:198 | 668:0:106 | 343:13:359 |
|  |  |  |  |  |  |  |  |  |  |  |
|  | cM | 16.6±1.2 | 7.2±1.1 | 12.9±1.3 | 17.0±1.1 | 11.3±1.0 | 32.1±2.0 | 12.3±0.9 | 6.8±0.6 | 25.6±1.7 |
|  |  |  |  |  |  |  |  |  |  |  |
|  | P* | .029 | .085 | .748 | .005 | .291 | <.0001 | .055 | .158 | <.0001 |

Map distances and standard errors (in centiMorgans; cM) were calculated from parental ditypes (PD), nonparental ditypes (NPD) and tetratypes (TT) according to Experimental Methods.

* P values indicate the probability that the observed distribution among PD:NPD:TT deviates from that expected for no interference due to chance (Stahl, 2008). P-values <0.05 indicate interference.
