# Supplementary material for: Pch2 Links Chromosome Axis Remodeling at Future Crossover Sites and Crossover Distribution during Yeast Meiosis
Source: PLoS Genet. 2009 Jul 24;5(7):e1000557. doi: 10.1371/journal.pgen.1000557 (PMC2708914; doi:10.1371/journal.pgen.1000557)
Supplement: Table S5 — S. cerevisiae strains used in this study. (0.04 MB DOC) [file pgen.1000557.s010.doc]

**Table S5.** *S. cerevisiae* strains used in this study.

Strain

Number Relevant genotype*

Recombination analysis on chromosomes III, VII, VIII

VBY1600 *MAT****a****/ MAT*α, *his4-B/ HIS4, leu2-R/ LEU2, CENIII::ADE2/ CENIII, CENVIII::URA3/ CENVIII, arg4-Bgl/ ARG4, thr1-A/ THR1, cup1 S/ CUP1, lys5-P/ LYS5, met13-B/ MET13, cyh2^R^/ CYH2^S^, trp5-S/ TRP5, ura3(Δsma-pst:hisG)/ ”, ade2Δ/ ”*(=mating of NHY1558 and NHY1673)

VBY1601 As VBY1600, except *pch2Δ::hphMX4/ ”*

Hypomorphic *spo11* strains

VBY1446 *MATa/ MATα, ade2 Δ/”, can1^R^/CAN1^S^, met13B/MET13, trp5-S/TRP5, CENVIII::URA3/CENVIII, CENIII/CENIII::ADE2, thr1-A/THR1, cup1^S^/CUP1, HIS4/his4-B, LYS5/lys5-P, CYH2/cyh2^R^, spo11-HA3His6::kanMX4/”* (mating of SKY633 and SKY635)

VBY1447 *MATa/MATα, ade2 Δ/”, can1^R^/CAN1^S^, met13B/MET13, trp5-S/TRP5, CENVIII::URA3/CENVIII, CENIII/CENIII::ADE2, thr1-A/THR1, cup1^S^/CUP1, HIS4/ his4-B, LYS5/ lys5-P, CYH2/cyh2^R^, spo11-HA3His6::kanMX4/ spo11-(Y135F)-HAHis6:: kanMX4* (mating of SKY633 and SKY665)

VBY1449 *MATa/MATα, ade2 Δ/”, can1^R^/CAN1^S^, met13B/MET13, trp5-S/TRP5, CENVIII::URA3/CENVIII, CENIII/CENIII::ADE2, thr1-A/THR1, cup1^S^/CUP1, HIS4/ his4-B, LYS5/lys5-P, CYH2/cyh2^R^, spo11-(D290A)-HAHis6:: kanMX4/”* (mating of SKY1062 and SKY638)

VBY1602 same as VBY1446, except *pch2Δ::hphMX4/”*

VBY1603 same as VBY1447, except *pch2Δ::hphMX4/”*

VBY1605 same as VBY1449, except *pch2Δ::hphMX4/”*

VBY1473 *MATa/MATα, his4X::LEU2-(BamHI)-URA3/HIS4::LEU2-(BamHI), spo11-(D290A)-HAHis6:: kanMX4/”,*

VBY1479 same as VBY1473 except *pch2Δ::hphMX4/”*

Cytology

VBY1317 *MATa/MATα, pch2-URA3::pPCH2(300bp):3xHA/”*

VBY1454 *MATa/MATα, ho::hisG/”, ZIP3-GFP::kanMX4/”, RED1-HA::URA3/”*

VBY1455 *MATa/MATα, ZIP3-GFP::kanMX4/”, pch2-URA3::pPCH2(300bp):3xHA/”*

VBY1457 *MATa/MATα, ZIP3-GFP::kanMX4/”*

VBY1456 *MATa/MATα, ZIP3-GFP::kanMX4/”, pch2Δ::kanMX4/”*

VBY1537 *MATa/MATα, dmc1Δ::kanMX4/”, ZIP3-GFP::kanMX4/”, RED1-HA::URA3/”*

VBY1544 *MATa/MATα, ndt80Δ::hphMX4/”, ZIP3-GFP::kanMX4/”*

VBY1545 *MATa/MATα, ndt80Δ::hphMX4/”, ZIP3-GFP::kanMX4/”, pch2Δ::kanMX4/”*

______________________________________________________________________________

* All strains are also *ura3::hisG/”, ho:hisG/”*
